# Supplementary material for: BIOPOINT: A particle-based model for probing nuclear mechanics and cell-ECM interactions via experimentally derived parameters
Source: PLoS Comput Biol. 2026 Mar 31;22(3):e1014113. doi: 10.1371/journal.pcbi.1014113 (PMC13056257; doi:10.1371/journal.pcbi.1014113)
Supplement: S1 Appendix — Fig A. Quantification of spreading by measuring projected surface area of cells. Fig B. Spatial stress distribution in the bottom surfaces of cells on patterns. Fig C: Drastic nuclear shape change in rectangular patterns. Fig D: Spatial strain analyses for cell migration through a constriction. Fig E: Potential between cytoplasmic and nuclear particles. Fig F: Evolution of shape in the cell with 20% nucleus -- 200/800 nuclear/cytoplasmic particles. Fig G: Force versus time for single cell indentation simulations with different values of Np. Fig H: Effect of immobilization of peripheral particles. Table A. Values of the LJ parameters and the corresponding cell height in cell spreading experiments. Table B. BIOPOINT rheological parameters and expected variation across cell types, with representative experimental ranges. (PDF) [file pcbi.1014113.s001.pdf]

BIOPOINT: A particle-based model for probing nuclear mechanics and cell-ECM interactions via experimentally derived parameters.

Sandipan Chattaraj[1,*], Julius Zimmermann[1], Francesco Silvio Pasqualini[1,*].

1. Dept of Civil Engineering and Architecture, University of Pavia: Universita degli Studi di Pavia, Pavia, ITALY

* To whom correspondence should be addressed

E-mail: francesco.pasqualini@unipv.it, sandi.chattaraj@gmail.com

Keywords: particle-based model, deformable nucleus, cell-ECM interactions, nuclear mechanics, single cell indentation

ABSTRACT

Morphogenesis arises from biochemical and biomechanical interactions across multiple spatial and temporal scales. Experimental studies alone cannot fully resolve these dynamics, motivating computational models. Subcellular element modeling (SEM) is well suited for simulating emergent cellular and tissue morphologies, but traditional SEM frameworks do not explicitly include nuclear deformation or direct cell–extracellular matrix (ECM) interactions: capabilities typically associated with continuum approaches based on the finite-element method (FEM) approaches, FEM excels at modeling cell and tissue mechanics, but struggle to accommodate the large, non-linear deformations driven by local, geometry-changing events that define morphogenesis.

Here, we introduce BIOPOINT, a particle-based framework that augments SEM with FEM-like mechanical capabilities by incorporating (1) a deformable, multi-particle nucleus capable of capturing nuclear stress and strain distributions and (2) an explicit ECM layer represented by structured static particles with tunable adhesive potentials. To ensure biological relevance, we calibrate BIOPOINT against single-cell indentation experiments (SKOV3). We then apply this calibrated parameter set, without additional refitting, to two independent scenarios: (i) cell (EC and hMSC) spreading on ECM micropatterns, capturing qualitative coupling between cell and nuclear shape; and (ii) confined migration (MDA-MB-231) through rigid constrictions, qualitatively reproducing the characteristic sequence of nuclear elongation and partial recovery. Differently from previous work, we present a full SEM model that uses heterogeneous particles to model nuclei, cells, and ECM via phase separation.

By combining SEM's strength in modeling emergent cell and tissue geometry with a mechanically sound handling of nuclear and ECM interactions, BIOPOINT provides a versatile platform for studying cell behaviors, like shape acquisition and migration through confinement that are relevant to morphogenesis. Implemented within the widely used, open-source LAMMPS ecosystem, BIOPOINT offers an accessible and extensible tool for the community.

SUPPLEMENTARY INFORMATION

1. Quantification of spreading by measuring projected surface area of cells

The degree of cell spreading on an ECM substrate depends on the strength of interaction between the cell and ECM. (Fig A-I). This can be quantified by measuring the projected surface area of the cell (Fig A-II). The steady state projected area after cell spreading is much higher for the strongly interacting cell-ECM.

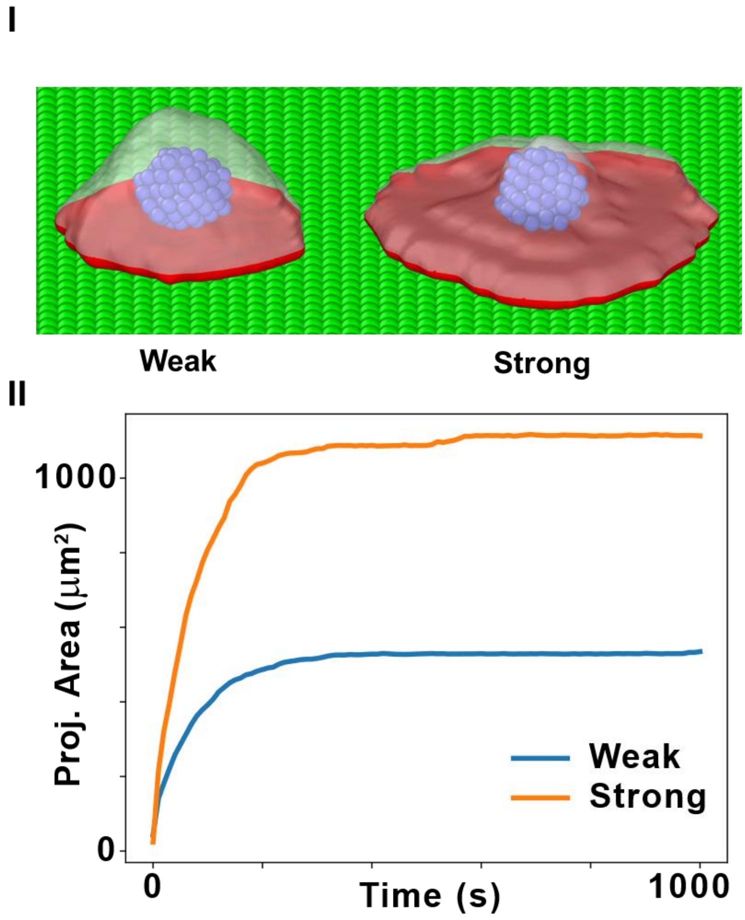

Fig A. Projected surface area of cells spreading on ECM. (I) Weak and strong interaction with extracellular matrix (ECM) particles can lead to different degrees of cell spreading. The projected surface area is indicated in red. II) Plot of the projected surface area of the cell as it spreads on ECM, versus time.

2. Spatial stress distribution in the bottom surfaces of cells on patterns

In the cell spreading simulations on circular (Fig B-I), square (Fig B-II) and triangular (Fig B-III) ECM patterns, mean volumetric stress per particle was computed utilizing LAMMPS "compute stress/atom" functionality. The particles and enclosing surfaces have been color coded as per the normalized mean volumetric stress. One can see on the bottom surface of the stretched-out cells that the peripheral region is under tensile (positive) stress whereas the inner region is under compressive (negative) stress.

There is a greater presence of positive/tensile stress on the peripheral particles and negative/compressive stress on the particles inside the cell. This is probably because the particles at the periphery are more stretched (from their equilibrium distances) than the ones in the interior, which are at relatively shorter distances. We have discussed this phenomenon in detail, in 3D in our previous publication [1].

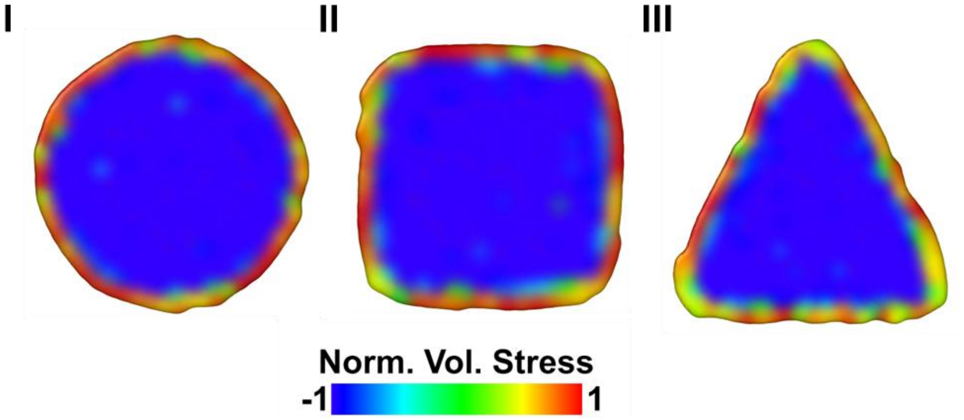

Fig B. Spatial stress distribution in the bottom surfaces of cells on patterns. Visualization of spatial distribution of normalized mean volumetric stress along the bottom surface of cells, which are spread on I) circular, II) square and III) triangular ECM patterns.

3. Drastic nuclear shape change in rectangular patterns

The experimental observation of drastic nuclear shape change in certain cell types such as the U2OS [2] could not be captured with our calibrated parameters obtained from the indentation simulations (Fig C-I). This could be because of the lack of an explicit treatment of actin filaments in our model. However, on including a potential between the nucleus and ECM particles (2-3 potential, Fig C-II), we observe that we could simulate scenarios of drastic shape change of the nucleus (Fig C-III). Though there is no direct connection between the ECM and the nucleus in reality, this might indicate an indirect connection between the nucleus and ECM in certain cell types where drastic nuclear shape change is observed during spreading. The cumulative effect of the actin filaments in these cell types might be captured through a 2-3 potential in our model.

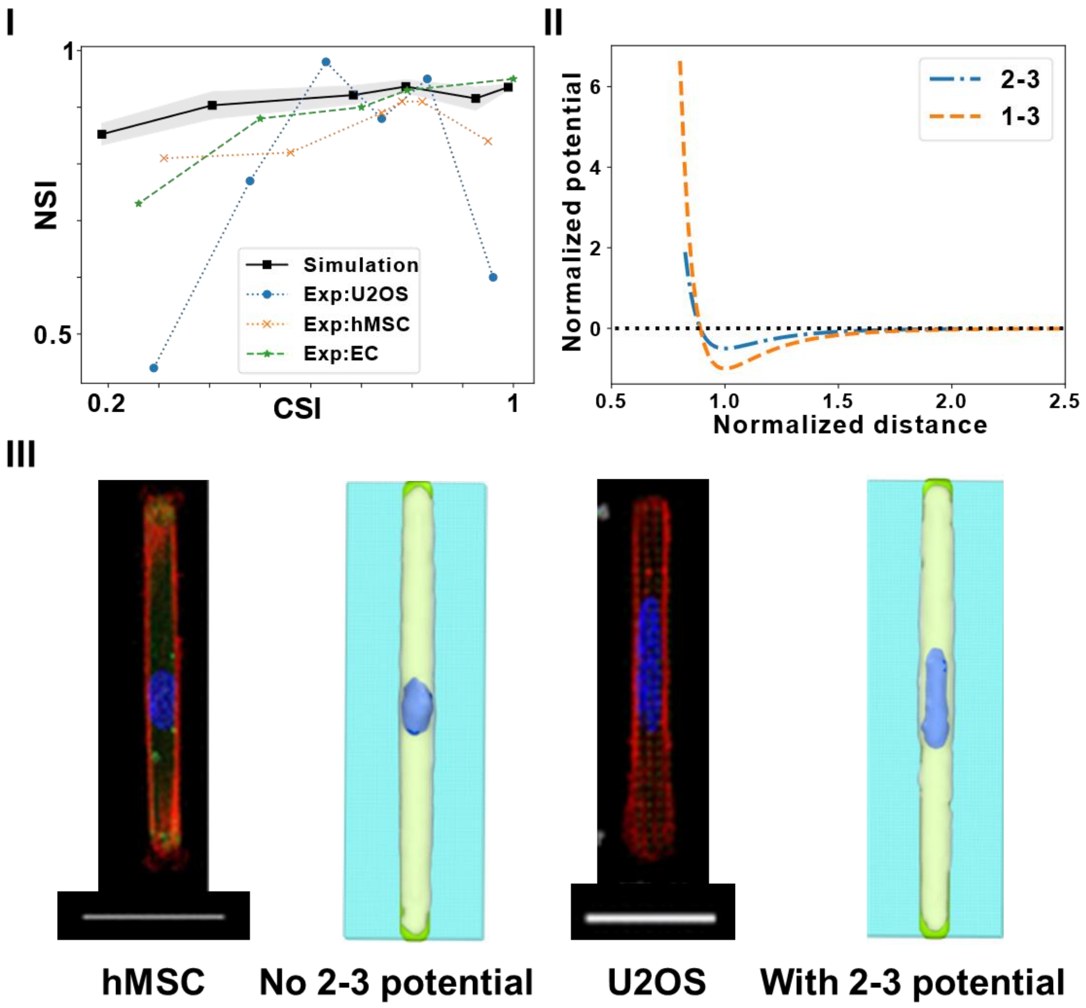

Fig C. Effect of the nuclear-ECM potential. I) Plot of NSI versus CSI for representative simulations and experimental results from the literature for cell types: hMSC, U2OS [2] and EC [3]. II) Plot of normalized potential (LJ 12-6) versus normalized distance for cytoplasm-ECM (1-3) and nucleus-ECM (2-3) interactions. III) Comparison of cell spreading simulations versus experimental results for hMSC and U2OS cells [2]. Experimental representative widefield images from Sarikhani *et al*. [2] have ECM in green, actin in red and nucleus in blue. Scale bar is 50 µm and 40 µm for hMSC and U2OS cells respectively. The simulations of cell spreading are without and with nucleus-ECM (2-3) potential, respectively.

4. Spatial strain analyses for cell migration through a constriction

As the cell moves through the narrow passage (Fig D-I, t_2), the nucleus elongates significantly (NSI decreases, ellipticity increases) due to external compression (Fig D-II). After exiting (t_3), the nucleus partially recovers its original shape but retains some deformation (Fig D-II), consistent with experimental observations. We leveraged BIOPOINT's particle-based approach, which allows spatial strain analysis even at the nuclear level. We computed per-particle shear and volumetric strain, revealing that the maximum shear strain occurs while the nucleus is inside the constriction (Fig D-III). Further, a residual strain persists even after passage, indicating incomplete shape recovery (Fig D-IV). Finally, a strong correlation exists between nuclear ellipticity and average strain, confirming that nuclear deformation is mechanically driven (Figs D-II, D-IV).

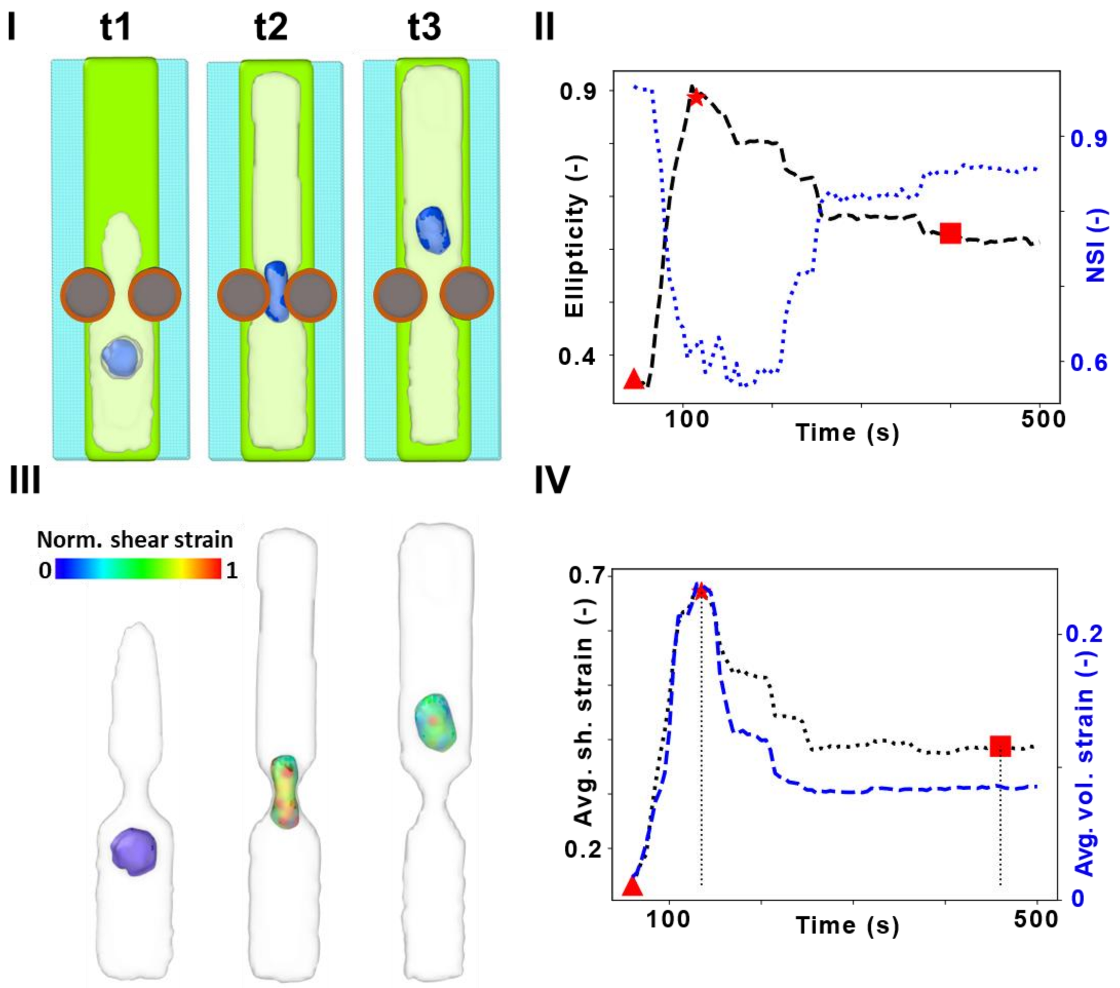

Fig D. Nuclear strain when cell passes through a narrow constriction. I) Simulated images of a nucleus changing shape at various time points, as a cell passes through a constriction. As in previous images, the ECM, cell membrane, nucleus and glass are represented by the colors green, white, blue and cyan, respectively. Brown circles are overlaid over the rigid constraints to enhance visualization. II) Nuclear ellipticity and NSI versus time as the cell passes through the constriction. III) Nucleus color-coded with shear strain shows how it is strained as it passes through the constriction. Cell membrane is depicted in white. IV) The mean shear and volumetric strain per nuclear particle go through a maximum as the nucleus pass through the constriction.

5. Potential between cytoplasmic and nuclear particles

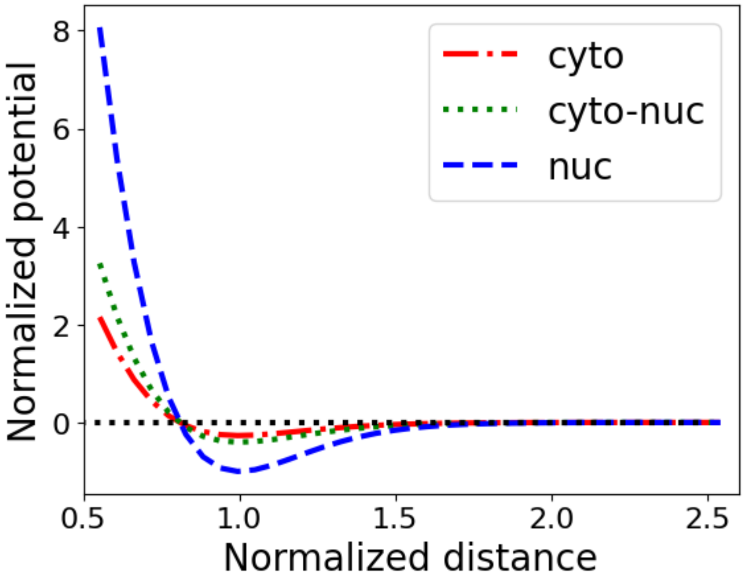

Fig E. Potential between cytoplasmic and nuclear particles. Plot of potential versus distance between cytoplasmic-nuclear particles along with cytoplasmic-cytoplasmic and nuclear-nuclear particles.

6. Evolution of shape in the cell with a larger (20%) nucleus

In order to investigate the effect of nuclear size, we have designed a cell with twice the nuclear size, i.e. 200 nuclear particles and 800 cytoplasmic particles. We have performed cell spreading on the various ECM patterns that were used in Fig. 3. We repeated the analyses of cell shape and nuclear shape for this cell with larger nucleus (Fig F). We didn't observe that the nucleus deforms much more than the smaller nucleus comprising 100 particles. This suggests that in the simulation scenarios studied here the potentials governing the nuclear, cytoplasmic and ECM particles and the interplay between them plays a bigger role in nuclear deformation than nuclear size.

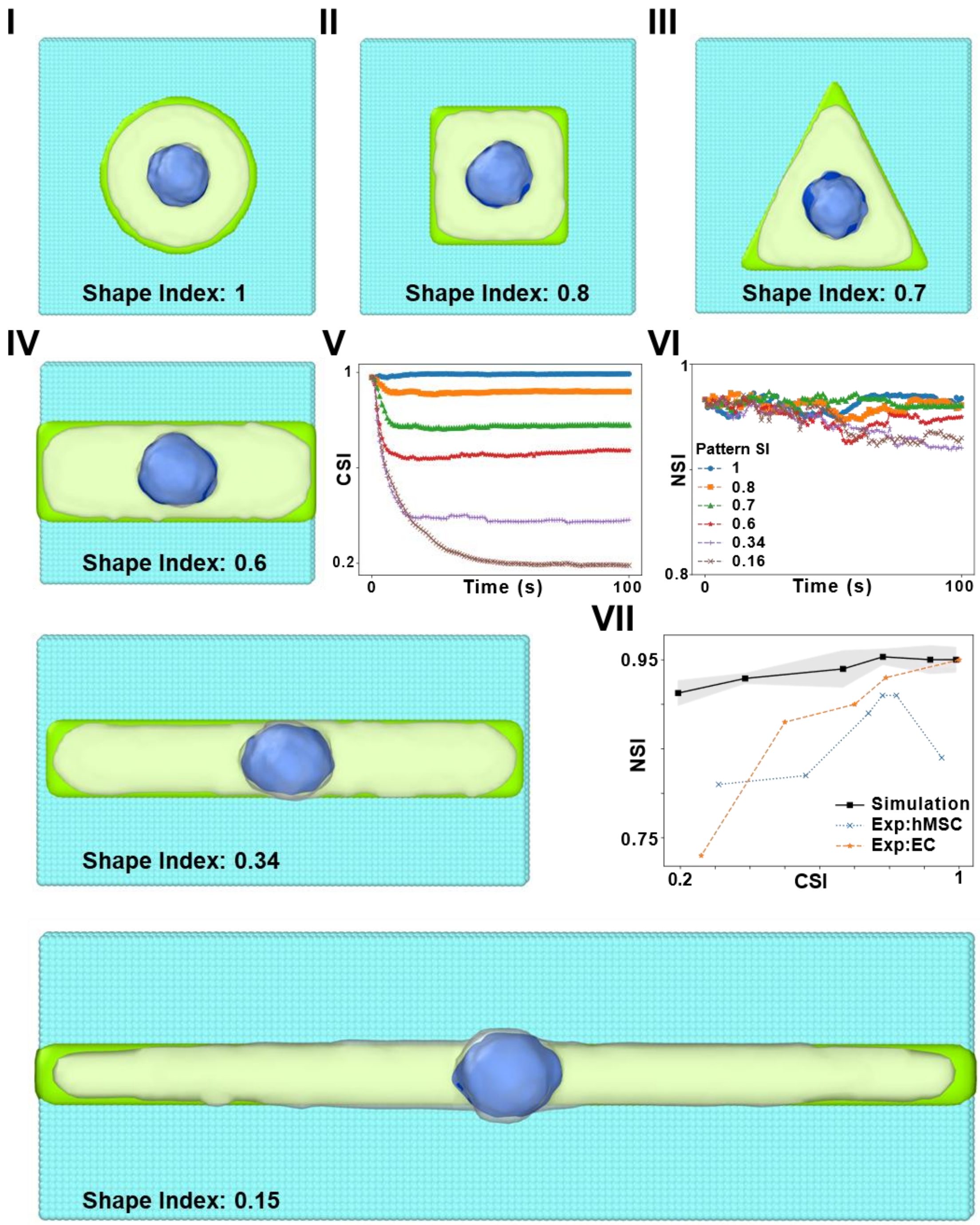

Fig F. Evolution of shape in the cell with 20% nucleus -- 200/800 nuclear/cytoplasmic particles. Simulations of cell spreading on circular (I), square (II), and triangular (III) ECM patterns, where the cell takes up the shape of the pattern. The cell membrane is depicted in white, the nucleus in blue, the ECM surface in green, and surrounding glass particles in cyan. IV) Cell spreading on three rectangular patterns with similar area but different shape index. V) Cell shape index (CSI) versus time as the cell spreads on the various ECM patterns. VI) Nuclear shape index (NSI) versus time during cell spreading on the various ECM patterns (legends of V and VI are the same). VII) Plot of NSI versus CSI for our simulations and experimental results from the literature: EC [3] and hMSC [2].

7. Determining the LJ potential parameters

The ECM particles act like a planar wall that attracts the cytoplasmic particles, resulting in cell spreading. In the scope of this work, there is no interaction between ECM particles. The LJ potential between cytoplasmic and ECM particles has been provided in Eq. 5 of the main manuscript. The attraction between cytoplasmic and ECM particles is modeled by a Lennard-Jones (12-6) potential:

$$V(d) = 4\varepsilon\left[\left(\frac{\sigma}{d}\right)^{12} - \left(\frac{\sigma}{d}\right)^{6}\right] \quad d < d_c$$

d is the distance between the cytoplasmic and ECM particles, σ is the zero-crossing distance, ε is the potential well-depth, and d_c is the cutoff distance. The gap between the planar ECM particles and the cytoplasmic particles is fixed for visualization purposes, such that σ = 5μm. This value can be tuned to adjust the gap between the ECM and the cell. The cut-off distance, d_c, is set as the sum of σ and the radius of the cell, so that there is interaction between the particles in the bottom hemisphere of the cell and the ECM -- d_c = 15μm. The potential well-depth, ε, has been varied such that the cell's height matches that of the cell in the AFM indentation experiment (Hobson et al., 2020). Table A provides the values for the LJ parameters and the corresponding cell height in cell-spreading experiments:

Table A. Values for the LJ parameters and the corresponding cell height in cell-spreading experiments.

σ(μm)	ε (zJ)	d_c(μm)	Cell Height (μm)
5	25000	15	13.9
5	50000	15	11.4
5	75000	15	11

In order to prepare the *'in silico'* cell for indentation simulations, we needed a more flattened cell and nucleus. We obtained the flattening with a nucleus-cytoplasm potential switched on with a well-depth half of that between cytoplasm-ECM. Once the desired cell height is achieved (~ 9 μm), the nucleus-cytoplasm potential was switched off and the cell was equilibrated again after spreading.

8. Varying the number of particles in a cell, N_p

In order to investigate the effect of the number of particles, we have varied the N_p slightly to 1200 and also drastically to 10000. The size of the nucleus remains 10% of the cell in both these scenarios. Ideally, this requires preparing a new cell with revised number of particles, phase separating the nucleus and recalibrating the parameters. This process can become computationally expensive with a drastic increase in the number of particles. We have prepared two new cells with N_p = 1200, 10000 and derived the geometric parameter values, such as d_{eq}, particle viscosity, stiffness from the scaling relations of Eq. 3. For the bulk properties of cell and nucleus, we have continued with the values that we derived from optimization with N_p = 1000. We performed single cell indentation on each of these cells and plotted the force-time curves (Fig. G). We found that the elasticity is qualitatively similar for both N_p =1200 and 10000, with similar peaks of the force-displacement curve at the end of the loading period of indentation. The force relaxation in the hold period varies more dramatically for N_p = 1200 and more so for N_p = 10000. We recommend that the particle number, and hence size, should be chosen carefully before using our model, depending on the length scale that one wishes to probe in their applications. If the particle number is changed, it is necessary to redo the phase separation, and recalibrate.

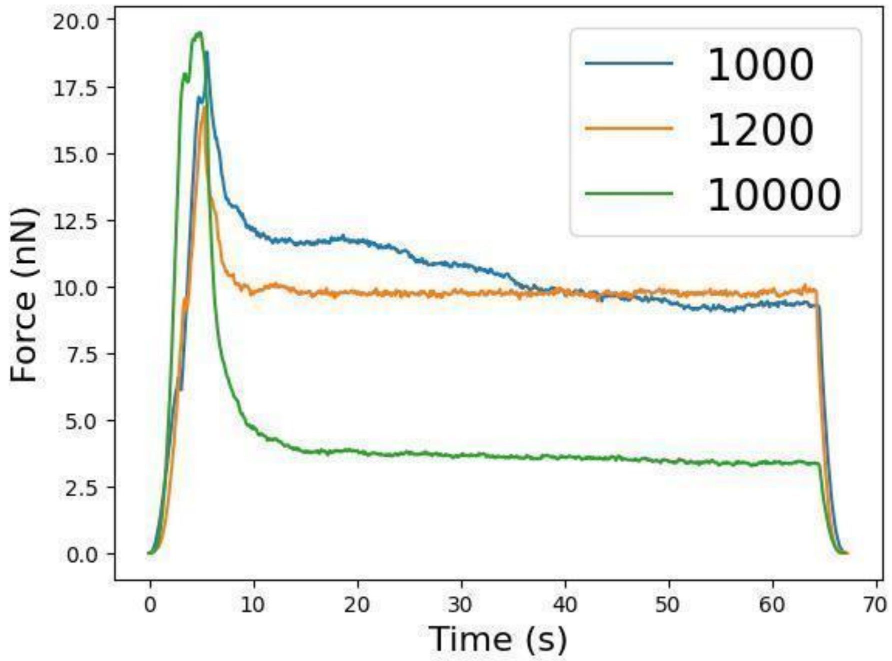

Fig G. Force versus time for single cell indentation simulations with different values of N_p.

9. Effect of immobilization of peripheral particles

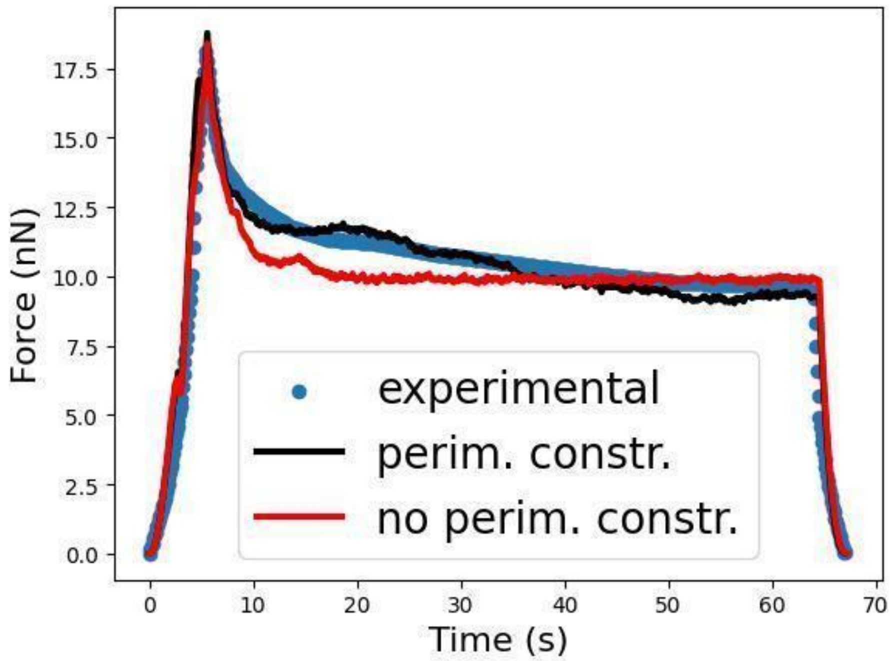

Fig H. Effect of immobilization of peripheral particles. Force versus time curve of indentation simulations with and without constraint on the peripheral particles.

10. Expected ranges of parameter values for different cell types

In BIOPOINT, the key rheological parameters are the cytoplasmic and nuclear spring constants (κ_0, κ_{nuc}) and viscosities (η_0, η_{nuc}) that define the pairwise interaction potentials between particles (Tables 1 and 2). These quantities are not direct continuum moduli, but were calibrated by matching AFM indentation force–time curves against SKOV3 cells using a Kelvin-Voigt-like viscoelastic response at the coarse-grained level. To estimate how these parameters would change for other cell types, we reviewed experimental measurements of apparent elastic moduli and viscosities for cytoplasm, cortex, and nuclei in representative mammalian cells.

Apparent cell stiffness. AFM-based indentation and nanorheology studies routinely find that adherent mammalian cells span roughly 1–2 orders of magnitude in apparent Young's modulus, depending on cell type, substrate, probe geometry, and loading rate. A high-throughput nanorheology analysis of NIH 3T3 fibroblasts reported apparent moduli $E_0 \approx 0.6$–1.3 kPa over a broad range of indentation velocities, with modest stiffening at higher speeds [4]. In contrast, AFM nanoindentation of vascular endothelial cells (bEnd.3) using a conical tip and viscoelastic Neo-Hookean modeling yielded effective elastic moduli in the 16–20 kPa range, again with some loading-rate dependence [5]. Reviews of AFM measurements across many cell lines support a typical range of ~0.1–2 kPa for many epithelial and cancer cells, with fibroblasts and endothelial cells often an order of magnitude stiffer when probed at the cortex [4]. These values define the continuum "target" behavior that BIOPOINT's κ_0 is designed to reproduce. Because κ_0 scales approximately linearly with the apparent indentation modulus for a fixed geometry, we expect κ_0 for other adherent cell types to vary by ~0.1–10× around the SKOV3-fitted value.

Nuclear stiffness. Multiple AFM and micromanipulation studies concur that the nucleus is typically stiffer than the surrounding cytoplasm and that its modulus is strongly tuned by lamin A/C and chromatin state. A recent study on live cells reported cytoplasmic stiffness of ~0.5–3 kPa and nuclear stiffness in the ~1–10 kPa range, depending on lamin distribution and substrate conditions. Direct AFM probing of exposed endothelial nuclei found nuclear regions with mean modulus ~5.6 kPa compared to ~1.5 kPa in non-nuclear regions of the same cells (nucleus/cytoplasm stiffness ratio ≈3–4) [6]. At the softer end, AFM measurements on MDA-MB-231 breast cancer cells showed nuclear stiffness values of order 10^2 Pa, which increased roughly twofold upon histone deacetylase inhibition, again preserving a nucleus-to-cytoplasm stiffness ratio of a few-fold [7]. These data imply that, across adherent mammalian cells, nuclear moduli occupy roughly 0.2–10 kPa, with a fairly robust nucleus/cytoplasm stiffness ratio of ~2–4. BIOPOINT's optimized parameters ($\kappa_{nuc} \approx 3.8\times\kappa_0$) fall squarely in this range, and we therefore expect κ_{nuc} to vary on the same order as κ_0 (roughly 0.2–10× its SKOV3 value) when targeting different cell types, while maintaining a stiffness ratio in the 2–4 band. This justifies the prior range 1–3 used for the coupling parameter k_{12} in our uncertainty quantification.

Cytoplasmic viscosity and relaxation times. Cytoplasmic viscosity has been quantified using a variety of techniques, including micropipette aspiration, particle-tracking microrheology, and magnetic rotational spectroscopy (MRS), and exhibits large variability across cell types and intracellular locations. MRS measurements in mammary epithelial cells (MCF-10A) and breast cancer lines (MCF-7, MDA-MB-231) reported cytoplasmic viscosities in the range 10–70 Pa·s with viscoelastic moduli of 30–80 Pa, yielding characteristic relaxation times $\tau = \eta/G$ on the order of 0.1–3 s [8]. A micropipette-based analysis of hundreds of individual lung tumor cells (H1299, A549) treated as Newtonian drops found much broader single-cell viscosity distributions: for H1299 cells, quartiles spanned ~17, 42, and 110 Pa·s, while A549 cells exhibited medians near 490 Pa·s with upper quartiles above 10^3 Pa·s [9]. Earlier work on white blood cells and embryonic systems reported cytoplasmic viscosities ranging from a few Pa·s up to several hundred Pa·s, depending on probe size and timescale [10]. Together, these data suggest an "operational" cytoplasmic viscosity range of ~1–10^3 Pa·s for many mammalian cells, with differences of 5–10× between related lines (e.g., low- vs high-metastatic cells) being common [11]. Nuclear viscosities are less well constrained experimentally, but chromatin-focused rheology and HP1α perturbation experiments consistently show that chromatin-rich nuclear regions are more dissipative than the surrounding cytoplasm, leading to relaxation times that are typically a few-fold longer [12]. In BIOPOINT, η_0 and η_{nuc} are tuned to match the AFM force–relaxation curve of SKOV3; the experimental ranges above imply that re-fitting η_0 and η_{nuc} for different cell types should mainly involve ~0.1–10× rescaling.

Link to BIOPOINT parameter ranges. The discrete spring and dashpot parameters in BIOPOINT (κ_0, η_0, κ_{nuc}, η_{nuc}) are not directly equal to continuum Young's moduli and viscosities, but for a fixed particle discretization and indentation geometry they scale approximately linearly with the corresponding apparent moduli. Our SKOV3 calibration targets AFM data that fall within the low-kPa and 10–100 Pa·s regimes discussed above [13]. Taken together, the literature therefore supports using prior ranges of roughly 0.1–10× the SKOV3-fitted values for both K and η when adapting BIOPOINT to other adherent mammalian cell types. More extreme phenotypes (very soft embryonic cells, highly stiffened myofibroblasts, etc.) may require excursions up to ~2 orders of magnitude, but even in those cases the nucleus/cytoplasm stiffness ratio and the qualitative ordering $\eta_{nuc} > \eta_0$ are expected

to hold. The ranges summarized in Table B can thus serve as biologically grounded priors or bounds when re-calibrating BIOPOINT for new cell lines or conditions.

Table B. BIOPOINT rheological parameters and expected variation across cell types, with representative experimental ranges.

BIOPOINT parameter	Physical interpretation	SKOV3-fitted value (this work)	Expected variation for other adherent mammalian cells*	Representative experimental ranges / examples (continuum quantities)	Key refs
κ_0 [N·m^{-1}]	Effective spring constant for cytoplasmic + cortical network, controlling cell indentation response	1.2×10^{-2} N·m^{-1}	~0.1–10× SKOV3 (≈10^{-3}–10^{-1} N·m^{-1})	Apparent cell Young's modulus E_cell ≈ 0.1–2 kPa for many epithelial/cancer cells; NIH 3T3 fibroblasts ~0.6–1.3 kPa; vascular endothelial cells up to ~16–20 kPa depending on loading rate.	[4,5]
η_0 [N·s·m^{-1}]	Effective viscous damping between cytoplasmic particles (Kelvin-Voigt–like dashpot)	5.0×10^{-2} N·s·m^{-1}	~0.1–10× SKOV3 (≈5×10^{-3}–5×10^{-1} N·s·m^{-1})	Cytoplasmic viscosities from microrheology/micropipette typically ~10–70 Pa·s for mammary epithelial/cancer cells (MCF-10A/MCF-7/MDA-MB-231), with single-cell distributions spanning ~0.6–500 Pa·s; lung cancer cells (H1299, A549) show medians from ~40 to ~500 Pa·s and upper quartiles above 10^3 Pa·s.	[8,9]

κ_{nuc} [N·m⁻¹]	Spring constant for nuclear phase, capturing nuclear stiffness	4.5×10^{-2} N·m⁻¹	0.2–10× SKOV3 (≈10^{-2}–5×10^{-1} N·m⁻¹)	Nucleus generally stiffer than cytoplasm: cytoplasmic moduli ~0.5–3 kPa vs nuclear moduli ~1–10 kPa in many cell types;[14] MDA-MB-231 nuclei can be as soft as ~0.1–0.2 kPa under some perturbations, while exposed endothelial nuclei reach ~5–6 kPa.[7]	[6,7,14]
η_{nuc} [N·s·m⁻¹]	Viscous damping within the nuclear phase	2.1×10^{-1} N·s·m⁻¹	~0.2–10× SKOV3 (≈4×10^{-2}–2 N·s·m⁻¹)	Cytoplasmic MRS measurements give η ≈ 10–70 Pa·s with elastic moduli 30–80 Pa, corresponding to relaxation times τ ≈ 0.1–3 s; nuclear or chromatin-dominated regions are typically a few-fold more viscous.[8]	[8,11,12]
k_{12} [–]	Relative strength of nucleus–cytoplasm interaction potential	1.51	Likely 1–3	AFM and FEA studies consistently report nucleus/cytoplasm stiffness ratios between ~2 and 4 in many adherent cell types.[14]	[14]
λ [–]	Dimensionless tuning coefficient	0.75	Expected near 0.5–1 for most cell types; primarily numerical/phenomenological rather than directly tied to a single rheological observable	No direct experimental analog.	BIOPOINT (this work)

*Order-of-magnitude estimates based on published ranges of apparent moduli and viscosities; precise values should be re-identified by re-fitting BIOPOINT to AFM/microrheology data for the specific cell type of interest.

REFERENCES

1. Chattaraj S, Torre M, Kalcher C, Stukowski A, Morganti S, Reali A, et al. SEM2: Introducing mechanics in cell and tissue modeling using coarse-grained homogeneous particle dynamics. APL Bioengineering. 2023;7: 046118. doi:10.1063/5.0166829

2. Sarikhani E, Meganathan DP, Larsen A-KK, Rahmani K, Tsai C-T, Lu C-H, et al. Engineering the Cellular Microenvironment: Integrating Three-Dimensional Nontopographical and Two-Dimensional Biochemical Cues for Precise Control of Cellular Behavior. ACS Nano. 2024;18: 19064–19076. doi:10.1021/acsnano.4c03743

3. Versaevel M, Grevesse T, Gabriele S. Spatial coordination between cell and nuclear shape within micropatterned endothelial cells. Nat Commun. 2012;3. doi:10.1038/ncomms1668

4. Garcia PD, Guerrero CR, Garcia R. Nanorheology of living cells measured by AFM-based force–distance curves. Nanoscale. 2020;12: 9133–9143. doi:10.1039/C9NR10316C

5. Wang L, Tian L, Zhang W, Wang Z, Liu X. Effect of AFM Nanoindentation Loading Rate on the Characterization of Mechanical Properties of Vascular Endothelial Cell. Micromachines. 2020;11: 562. doi:10.3390/mi11060562

6. Wang K, Qin Y, Chen Y. In situ AFM detection of the stiffness of the in situ exposed cell nucleus. Biochimica et Biophysica Acta (BBA) - Molecular Cell Research. 2021;1868: 118985. doi:10.1016/j.bbamcr.2021.118985

7. Fischer T, Hayn A, Mierke CT. Effect of Nuclear Stiffness on Cell Mechanics and Migration of Human Breast Cancer Cells. Front Cell Dev Biol. 2020;8: 393. doi:10.3389/fcell.2020.00393

8. Dessard M, Manneville J-B, Berret J-F. Cytoplasmic viscosity is a potential biomarker for metastatic breast cancer cells. Nanoscale Adv. 2024;6: 1727–1738. doi:10.1039/D4NA00003J

9. Wang K, Sun XH, Zhang Y, Zhang T, Zheng Y, Wei YC, et al. Characterization of cytoplasmic viscosity of hundreds of single tumour cells based on micropipette aspiration. R Soc open sci. 2019;6: 181707. doi:10.1098/rsos.181707

10. Daniels BR, Masi BC, Wirtz D. Probing Single-Cell Micromechanics In Vivo: The Microrheology of C. elegans Developing Embryos. Biophysical Journal. 2006;90: 4712–4719. doi:10.1529/biophysj.105.080606

11. Moeendarbary E, Valon L, Fritzsche M, Harris AR, Moulding DA, Thrasher AJ, et al. The cytoplasm of living cells behaves as a poroelastic material. Nature Mater. 2013;12: 253–261. doi:10.1038/nmat3517

12. Strom AR, Biggs RJ, Banigan EJ, Wang X, Chiu K, Herman C, et al. HP1α is a chromatin crosslinker that controls nuclear and mitotic chromosome mechanics. eLife. 2021;10: e63972. doi:10.7554/eLife.63972

13. Hobson CM, Kern M, O'Brien ET, Stephens AD, Falvo MR, Superfine R. Correlating nuclear morphology and external force with combined atomic force microscopy and light sheet imaging separates roles of chromatin and lamin A/C in nuclear mechanics. Discher D, editor. MBoC. 2020;31: 1788–1801. doi:10.1091/mbc.E20-01-0073

14. Srivastava LK, Ju Z, Ghagre A, Ehrlicher AJ. Spatial distribution of lamin A/C determines nuclear stiffness and stress-mediated deformation. Journal of Cell Science. 2021;134: jcs248559. doi:10.1242/jcs.248559
