# Supplementary material for: BIOPOINT: A particle-based model for probing nuclear mechanics and cell-ECM interactions via experimentally derived parameters
Source: PLoS Comput Biol. 2026 Mar 31;22(3):e1014113. doi: 10.1371/journal.pcbi.1014113 (PMC13056257; doi:10.1371/journal.pcbi.1014113)

We thank the editors and the reviewers for their work in improving our manuscript. We present below a detailed response to the received feedback, as well as a summary of the modifications implemented in the manuscript.

Additional Editor Comments:

Comment

First, I would like to apologize for the delay in handling this manuscript. We had an exceptionally difficult time securing reviewers, likely due to the time of year. Ultimately, I agree with the reviewer concerns about the manuscript's clarity, especially regarding parameter definitions and model presentation. I also like to raise a concern about the framework's novelty. It remains unclear what BIOPOINT provides that hasn't already been addressed by previous continuum-based methods. For instance, Estabrook et al. (2021), who reconstruct nuclear deformation forces from experimental images using elastic solid and shell models, and Maxian et al. (2020), who modeled cell–ECM–nucleus mechanics including hydrodynamics in a FEM setting. Without a clear advantage over these established approaches, the novelty of BIOPOINT appears limited to a different computational implementation rather than a conceptual advance. If the authors believe there is indeed a novel advantage to this framework that is otherwise unobtainable through these other methods, and can convincingly rewrite the manuscript to convey and demonstrate this, the manuscript may be considered for publication. However, until then, I cannot recommend it for such. [...]

Response

We understand these are challenging times to secure reviewers for the manuscript, and we wish to thank you again for all your efforts. We have addressed the methodological issues throughout the manuscript, as you'll see below. Here, we'd like to clarify the novelty aspects, which is better argued for in the new Abstract and Discussions. Finite element analysis (FEA) and all its different implementations are a fantastic technology that remains the state-of-the-art for many/most situations. However, in scenarios where local events drive large non-linear deformations, e.g., tissue morphogenesis, SEM provides an attractive alternative option for its ability to treat shape as an emergent property of particle interaction. In this context, we sought to improve existing SEM models by incorporating nuclear deformation capabilities. In other words, we are not trying to beat FEA here; instead, we are trying to provide similar capabilities to SEM so that they can be employed in the context of morphogenetic systems.

To this end, it's worth mentioning here (as we also did in the revised introduction of the manuscript) that Reviewer 1 mentioned another paper where a SEM model had nuclear deformation capabilities. Reviewer 1 mentioned it as Kumar, 2024, but probably intended a paper from the same group Nematbakhsh, 2020 https://journals.plos.org/ploscompbiol/article?id=10.1371/journal.pcbi.1008105. However, that manuscript leverages a hybrid approach in which the nucleus is modeled with particles (so SEM) while cells and ECM are treated as a lattice of interconnected springs at the boundary of the cells (similar to vertex modeling). Moreover, they used a custom code that isn't widely accessible. We take this as evidence for interest in the implementations of discrete models capable of addressing nuclear deformation, while resting our novelty claim on the phase-separated nuclear and ECM particles treatment obtained as part of the widely accepted and community-maintained LAMMPS library. While an implementation of morphogenetic events with BIO-POINT was out-of-scope in this manuscript, we believe the open-source nature of our program will facilitate a more rapid adoption of SEM than previous efforts.

Modifications.

We rewrote the abstract as follows:

- "Morphogenesis arises from biochemical and biomechanical interactions across multiple spatial and temporal scales. Experimental studies alone cannot fully resolve these dynamics, motivating computational models. Subcellular element modeling (SEM) is well suited for simulating emergent cellular and tissue morphologies, but traditional SEM frameworks do not explicitly include nuclear deformation or direct cell–extracellular matrix (ECM) interactions: capabilities typically associated with continuum approaches based on the finite-element method (FEM) approaches, FEM excels at modeling cell and tissue mechanics, but struggle to accommodate the large, non-linear deformations driven by local, geometry-changing events that define morphogenesis.

  Here, we introduce BIOPOINT, a particle-based framework that augments SEM with FEM-like mechanical capabilities by incorporating (1) a deformable, multi-particle nucleus capable of capturing nuclear stress and strain distributions and (2) an explicit ECM layer represented by structured static particles with tunable adhesive potentials. To ensure biological relevance, we calibrate BIOPOINT against single-cell indentation experiments. We then validate the framework across three independent scenarios: (i) single-cell indentation with force–time responses matching atomic force microscopy data; (ii) cell spreading on ECM micropatterns, where nuclear deformation follows ECM geometry; and (iii) confined migration, where BIOPOINT reproduces nuclear shape dynamics as cells traverse constrictions. Differently from previous work, we present a full SEM model that uses heterogeneous particles to model nuclei, cells, and ECM via phase separation.

  By combining SEM's strength in modeling emergent cell and tissue geometry with a mechanically sound handling of nuclear and ECM interactions, BIOPOINT provides a versatile platform for studying cell behaviors, like shape acquisition and migration through confinement that are relevant to morphogenesis. Implemented within the widely used, open-source LAMMPS ecosystem, BIOPOINT offers an accessible and extensible tool for the community."

We also update the introduction as follows:

- After the sentence:
  "As a result, current SEM frameworks cannot accurately model processes such as nuclear deformation during confined migration or how ECM geometry influences cell spreading and mechanosensing."

  We added:
  "By contrast, continuum finite-element approaches can reconstruct nuclear stress–strain fields and cell–ECM coupling at the single-cell level (e.g., Estabrook et al., 2021; Maxian et al., 2020), but they rely on prescribed meshes and boundary conditions and are not easily embedded into large-scale morphogenetic simulations with large deformations, neighbor exchanges, and topological changes. Our goal here is therefore not to replace finite-element models, but to extend SEM so that these same classes of nuclear and cell–ECM mechanics can be addressed in a discrete, particle-based framework where cell and nuclear shape emerge from local interactions."

- Before the sentence:
  "To address these limitations, we propose two advancements."

  We added:

"Discrete models naturally capture biological processes but are costly, and cell mechanics is lost (e.g., Potts models [23]) or limited to cell membranes (e.g., vertex modeling [7]). Yet, the mechanical forces at play in the intracellular space, or cytoplasm, can influence the mechanical behavior of entire tissues [24,25]. Therefore, SEM remains the discrete modeling framework with the advantage of being able to model cell and tissue shapes as emergent properties of subcellular interactions [22,26]. Yet, it lacks the ability to handle the cell nucleus and the ECM as heterogeneous particle types. Previous discrete approaches have modeled a deformable nucleus using particles coupled to lattice-based descriptions of the cell and ECM in custom codes (e.g., Nematbakhsh et al., 2020 [22]), underscoring the demand for such capabilities but leaving a gap for a fully SEM-based, open-source implementation that treats the cell, nucleus, and ECM within a single particle framework."

- After the original final sentence:

  "This approach aims to demonstrate that BIOPOINT extends to the nucleus and the ECM the ability of SEM to simulate emergent shape and mechanical changes, filling a gap in morphogenesis modeling."

  We added:

  "In doing so, BIOPOINT complements established continuum frameworks by making experimentally calibrated nuclear and ECM mechanics available within a community-maintained LAMMPS implementation, lowering the barrier to deploying SEM in realistic morphogenetic simulations rather than merely offering an alternative numerical discretization."

- Furthermore, we have included a background result section covering the fundamentals of SEM before introducing our new framework.

  Theory of SEM: Simulating cells and tissues with SEM implies solving the Langevin equation for an arbitrary number of subcellular particles (N_p)[16].

$$\eta \dot{y}_{i,j,k} = \xi_{i,j,k} + F_C(y_{i,j,k}) \qquad (1)$$

In Eq. (1), η is the viscous drag coefficient and $y_{i,j,k}$, $\dot{y}_{i,j,k}$ are the position and velocity of each particle. We chose the indexes i, j, k to denote particle number, particle type, and cell number, respectively. With this notation, $\xi_{i,j,k}$ is the thermal fluctuation felt by each particle and $F_C(y_{i,j,k})$ is the net pairwise force acting on it. In particular, Newman used the following Morse-like elastic potential ($V(d)$ where d is the inter-particle distance) to enforce adhesion and volume exclusion (Fig 1B and Eq. 2) [16,18].

$$V(d) = u_0 e^{2\rho\left(1-\frac{d^2}{d_{eq}^2}\right)} - \alpha u_0 e^{\rho\left(1-\frac{d^2}{d_{eq}^2}\right)} \qquad (2)$$

In Eq. (2), u_0 is the potential's well depth, ρ and α are scaling and shifting factors, and d_{eq} is the equilibrium distance between particles. To limit the computational cost, these potentials focus on short-range interactions and cut off at 2.5 times the equilibrium distance. To assign the forces in Eq. (1) based on cell-level rheology, Newman assumed N_p densely packed particles connected by springs and recovered the following relationships[16]

$$d_{eq} = 2R_{cell}\left(\frac{p_d}{N_p}\right)^{\frac{1}{3}} \qquad (3a)$$

$$\eta = \frac{\eta_0}{N_p} \quad (3b)$$

$$\kappa = \kappa_0 N_p^{-\frac{1}{3}}(1 - \lambda N_p^{-\frac{1}{3}}) \quad (3c)$$

$$u_0 = \kappa \frac{d_{eq}^2}{(8\rho^2)} \quad (3d)$$

In Eqs. (3a-3d), p_d is the sphere close packing density, R_{cell}, is the radius of the cell, κ_0 and η_0 are the stiffness and viscosity of the cytoplasm, respectively. λ is a tuning coefficient which is an adjustment to take care of the departure from a cubic lattice arrangement used to derive the elastic modulus of the network [16]. The list of parameters and their values are listed in Table 1. We have inherited some of the basic parameter values, related to the functional form of the potential, such as the scaling factor, shifting factor, tuning coefficient from an earlier SEM model [18]. The model is calibrated on a particular cell type and ideally it should be applied on that same cell type and similar experimental conditions for quantitative match with experiments.

Table 1: Parameters used in SEM²

Parameter	Description	Value
R_{cell}	Cell radius	10 μm
m_{cell}	Cell mass	3.1 ng
N_p	Total number of particles in a cell	1000
p_d	Sphere close packing density	0.74
ρ	Scaling factor	2
α	Shifting factor	2
κ_0	Cell stiffness	1.2×10^{-2} N m^{-1}
η_0	Cell viscosity	5×10^{-2} N s m^{-1}
λ	Tuning coefficient	0.75
m_p	Mass of each particle	3.1×10^{-3} ng

- In Results, in the subsection, "BIOPOINT uses heterogeneous discrete particle types to model the cell nucleus and ECM", after the sentence:

  "These particles were assigned a stronger self-interaction potential (nuclear-nuclear adhesion) than cytoplasmic particles to induce phase separation."

  We added:

  "If κ_{nuc} and η_{nuc} are respectively the stiffness and viscosity of the nucleus, then the relative stiffness of cytoplasmic-nuclear interaction, in comparison to cytoplasmic stiffness is represented by the parameter, k_{12}.

$$k_{12} = \frac{u_0(1-2)}{u_0(1-1)} \quad (4)$$

  $u_0(1-2)$ and $u_0(1-1)$, respectively, represent potential well depth for the cytoplasm-ECM and cytoplasm-cytoplasm particle interactions, 1 and 2 representing particle type, as denoted by the index j in Eq. (1)."

- In Results, in the subsection, "BIOPOINT uses heterogeneous discrete particle types to model the cell nucleus and ECM", after the sentence:

  "These ECM particles exerted tunable adhesive interactions with cytoplasmic particles via a Lennard-Jones (LJ) potential."

  We added

  "The attraction between cytoplasmic and ECM particles is modeled by a Lennard-Jones (12-6) potential:

  $$V(d) = 4\varepsilon\left[\left(\frac{\sigma}{d}\right)^{12} - \left(\frac{\sigma}{d}\right)^{6}\right] \quad d < d_c \qquad (5)$$

  d is the distance between the cytoplasmic and ECM particles, σ is the zero-crossing distance, ε is the potential well-depth, and d_c is the cutoff distance. Details about the determination of LJ potential parameters is provided in SI. Table 2 lists the additional parameters needed in BIOPOINT to address multi-particle nucleus and cell-ECM interactions."

- We added Table 2:

Table 2: Additional parameters used in BIOPOINT

Parameter	Description	Value
N_n	Number of nuclear particles	100
κ_{nuc}	Nuclear stiffness	4.5×10^{-2} N m^{-1}
η_{nuc}	Nuclear viscosity	2.1×10^{-1} N s m^{-1}
k_{12}	Relative strength of cytoplasmic-nuclear potential	1.51
σ	Cell-ECM potential: zero-crossing distance	5 µm
ε	Cell-ECM potential: potential well-depth	60000 zJ
d_c	Cell-ECM potential: cutoff distance	15 µm

Reviewers' comments:

Reviewer's Responses to Questions

Reviewer #1

Comment 1.0. The authors present BIOPOINT, a subcellular-element model (SEM) type cell-based modelling framework based on the molecular dynamics software LAMMPS. BIOPOINT is a new iteration of previous SEM software SEM2 and SEM++. In this manuscript, the software has been extended to represent the cell nucleus with multiple particles and a two-dimensional representation of the extracellular matrix has also been implemented. Additionally, the authors calibrate the parameters of their model with previously-published mechanobiological experiments including atomic force microscopy microindentation and cell migration in confinement.
Development and experimental validation of better modelling software is important for the computational biology community, for which this manuscript has value. The authors also demonstrate that BIOPOINT can be used to determine internal stresses and strain in the cytosol and in the nucleus, a novel mechanobiological model prediction possible with this model that could be used to explore nuclear mechanics and chromatin organization. I have some questions and comments to the authors.

Response 1.0. We appreciate the reviewer's summary of our work and the provided inputs, which we have incorporated and made appropriate changes to the manuscript and SI.

Modification 1.0. No modifications.

Comment 1.1 The abstract states that "traditional SEM frameworks lack explicit representation of nuclear mechanics and cell-extracellular matrix (ECM) interactions". There are previous SEM works that simulate tissue mechanics that explicitly represent a multi-particle nucleus and extracellular matrix (for a recent example see Kumar et al. npj Systems Biology and Applications 10.1 (2024): 49.). Hence this statement in the abstract is not correct and should be revised.

Response 1.1

We clarified our claim that traditional SEM frameworks lack explicit representation of nuclear mechanics and cell-ECM interactions. A previous publication exists from the team of Jeremiah Zartman and Mark Alber, which reported a multi-particle nucleus and explicit ECM particles, along with membrane particles. We believe the paper was not the suggested Kumar et al. in NPJ Systems Biology and Applications, 2024 but rather the article by Nematbakhsh et al. in PLOS Computational Biology, 2020. The article is impressive and it further supports our claim that combining discrete particle mechanics is critical in modeling morphogenesis. However, as can be seen by their Figure 10 (reported below for convenience), they used a hybrid approach to model nuclei with SEM and the membrane and ECM particles with discrete spring connections, more similar to a vertex modeling approach than SEM. This approach is computationally lighter but loses spatial resolution of intracellular stress and strain as it lumps intracellular mechanics into discrete spring-like contributions We reworded our text to make the claim more accurate.

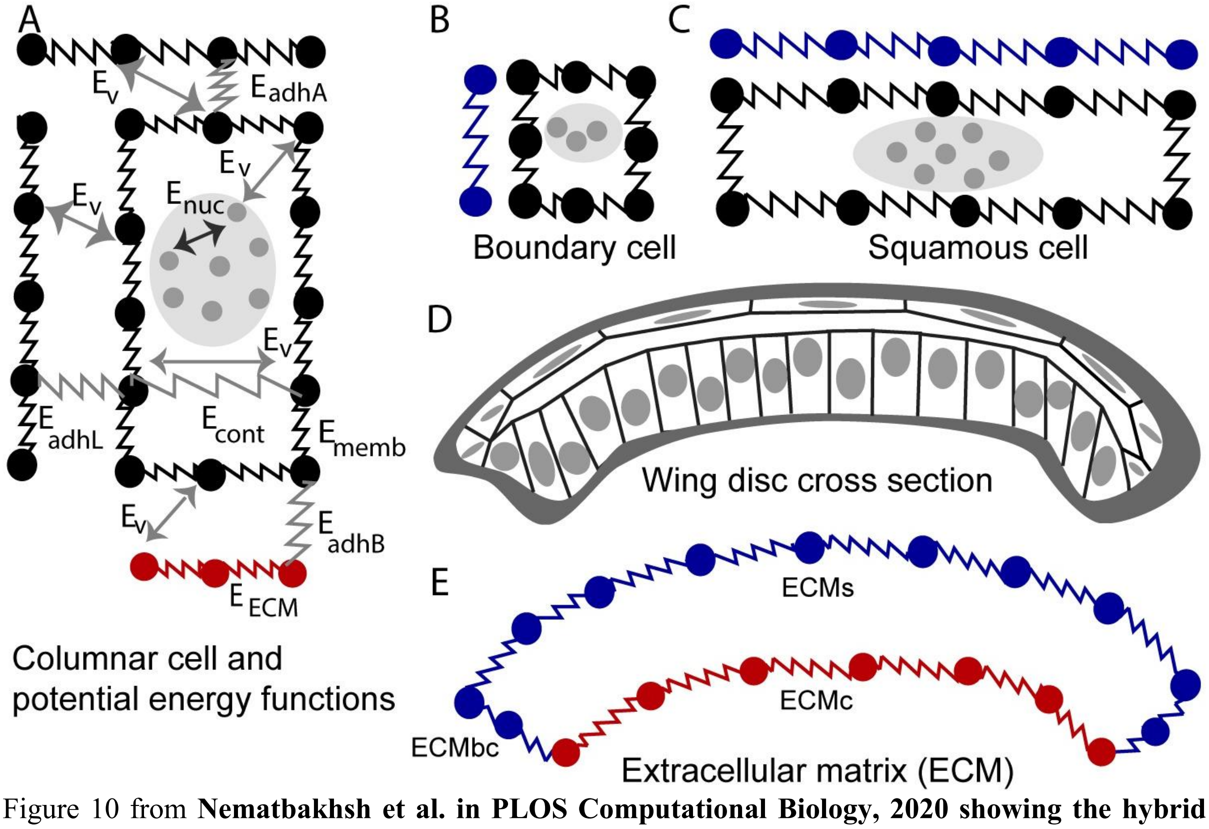

Figure 10 from **Nematbakhsh et al. in PLOS Computational Biology, 2020 showing the hybrid SEM (nucleus) and vertex modeling (cell membrane and ECM) simulation that's different from our full SEM approach.**

Modification 1.1

The following is the modified sentence in the abstract:

"Notably, prior works with the SEM frameworks have included nuclear deformation and ECM mechanics in morphogenetic processes, but used a hybrid approach that combined SEM for nuclear deformation, and interactions like cortical springs in the cell membrane and ECM. Instead, we present a full SEM model that uses heterogeneous particles to model nuclei, cells, and ECM via phase separation."

Comment 1.2. Related to the previous point, the introduction should provide better contextualization of prior work. The authors briefly mention and dismiss other modelling frameworks such as vertex and cellular Potts models. This is disappointing, as each framework has its merits and has been used to successfully simulate mechanobiology. In the authors' own previous work (Chattaraj et al. APL bioengineering 7.4 (2023).), they do a much better job at describing the broader context of cell based models including citing relevant reviews (Metzcar et al. JCO clinical cancer informatics 2 (2019): 1-13.) and previous relevant SEM work (e.g. Nematbakhsh et al. PLoS computational biology 16.8 (2020): e1008105.).
In addition, some other relevant papers the authors should acknowledge include work parametrizing cell based models to tissue mechanics (Pathmanathan et al. Physical biology 6.3 (2009): 036001.) and work modelling nuclear deformation during cell migration (Scianna and Preziosi. Axioms 10.1 (2021): 32.).

Response 1.2
We recognized that we had sacrified clarity for brevity in our original formulation, we have now added text discussing the merits and demerits of various frameworks along with mentioning the advantages and disadvantages of our framework.

Modification 1.2
The text in the Introduction has been changed from:
"Computational models provide a powerful means of integrating experimental data and predicting emergent behaviors that are otherwise difficult to capture in vitro [12]. While classical FEM approaches have provided detailed nuclear stress-strain predictions, they typically struggle with large cell deformations and tissue heterogeneity [13,14]. Vertex and Cellular Potts Models (CPM) can simulate migration but have lacked realistic cell and nuclear mechanics [15]. Subcellular Element Modeling (SEM) has emerged as a promising framework for simulating multicellular and subcellular mechanics [16–18]. SEM enables the modeling of emergent shapes by treating cells as ensembles of coarse-grained particles interacting via empirically defined potentials. Recently, we introduced SEM^2, which allows the calculation of multiscale mechanics in SEM simulations by assessing stress and strain at the particle, cell, and tissue levels [19]. However, a key limitation of existing SEM models is their inability to capture nuclear mechanics and cell-ECM interactions explicitly. Prior SEM implementations have typically represented the nucleus as a single rigid particle, which fails to account for nuclear deformability and the role of intracellular forces in nuclear positioning and chromatin remodeling. Similarly, the ECM has generally been modeled as an implicit solvent rather than a discrete, separate environment directly interacting with cells. Finally, SEM parameters were traditionally derived by homogenizing bulk elastic stiffness and viscosity of non-adherent cells, leading to model parameters that might not be relevant for cell-ECM interactions during morphogenesis. As a result, current SEM frameworks cannot accurately model processes such as nuclear deformation during confined migration or how ECM geometry influences cell spreading and mechanosensing. To address these limitations [...]"

To:

"Computational models provide a powerful means of integrating experimental data and explaining emergent behaviors [12]. While classical FEM approaches have provided detailed nuclear stress-strain predictions, they typically struggle with large cell deformations and tissue heterogeneity [13,14]. Vertex and Cellular Potts Models (CPM) can simulate large deformation and local events but struggle with representing distributed cell and nuclear mechanics [15]. Subcellular Element Modeling (SEM) has emerged as a promising framework for simulating multicellular and subcellular mechanics [16–18]. SEM enables the modeling of emergent shapes by treating cells as ensembles of coarse-grained particles interacting via empirically defined potentials. Recently, we introduced SEM^2, which allows the calculation of multiscale mechanics in SEM simulations by assessing stress and strain at the particle, cell, and tissue levels [19]. However, a key limitation of existing SEM models is their inability to capture nuclear mechanics and cell-ECM interactions explicitly. Prior SEM implementations have typically represented the nucleus as a single rigid particle, which fails to account for nuclear

deformability and the role of intracellular forces in nuclear positioning and chromatin remodeling. Similarly, the ECM has generally been modeled as an implicit solvent rather than a discrete, separate environment directly interacting with cells. Finally, SEM parameters were traditionally derived by homogenizing bulk elastic stiffness and viscosity of non-adherent cells, leading to model parameters that might not be relevant for cell-ECM interactions during morphogenesis. As a result, current SEM frameworks cannot accurately model processes such as nuclear deformation during confined migration or how ECM geometry influences cell spreading and mechanosensing. By contrast, continuum finite-element approaches can reconstruct nuclear stress–strain fields and cell–ECM coupling at the single-cell level (e.g., Estabrook et al., 2021; Maxian et al., 2020), but they rely on prescribed meshes and boundary conditions and are not easily embedded into large-scale morphogenetic simulations with large deformations, neighbor exchanges, and topological changes. Our goal here is therefore not to replace finite-element models, but to extend SEM so that these same classes of nuclear and cell–ECM mechanics can be addressed in a discrete, particle-based framework where cell and nuclear shape emerge from local interactions.

Discrete models naturally capture biological processes but are costly, and cell mechanics is lost (e.g., Potts models [23]) or limited to cell membranes (e.g., vertex modeling [7]). Yet, the mechanical forces at play in the intracellular space, or cytoplasm, can influence the mechanical behavior of entire tissues [24,25]. Therefore, SEM remains the discrete modeling framework with the advantage of being able to model cell and tissue shapes as emergent properties of subcellular interactions [22,26]. Yet, it lacks the ability to handle the cell nucleus and the ECM as heterogeneous particle types. Previous discrete approaches have modeled a deformable nucleus using particles coupled to lattice-based descriptions of the cell and ECM in custom codes (e.g., Nematbakhsh et al., 2020 [22]), underscoring the demand for such capabilities but leaving a gap for a fully SEM-based, open-source implementation that treats the cell, nucleus, and ECM within a single particle framework. To address these limitations, [...]”

Comment 1.3. The authors should provide a better description of what their model entails, which is lacking. I realize there is a prior publication that this model builds on, but at least a high-level summary would be needed. I notice also that equation numbering starts at 4 in the methods. Are equations 1-3 missing from the text?

Response 1.3
We have incorporated a subsection called "Theory of SEM" after the Introduction and before the Results. A theoretical description and a summary of the model is provided in this subsection. Additionally, we have brought Table 1 into the Theory of SEM section to give the readers an overview of the model's parameters and their values. We also thank the reviewers for pointing out the error in Equation numbering, which indeed occurred because of our dilemma in adding a theory section (which would mean repeating information from our previous publication). We have fixed it now.

Modification 1.3
We added the subsection, titled "Theory of SEM" after the Introduction.

Theory of SEM: Simulating cells and tissues with SEM implies solving the Langevin equation for an arbitrary number of subcellular particles (N_p)[16].

$$\eta \dot{y}_{i,j,k} = \xi_{i,j,k} + F_C(y_{i,j,k}) \qquad (1)$$

In Eq. (1), η is the viscous drag coefficient and $y_{i,j,k}$, $\dot{y}_{i,j,k}$ are the position and velocity of each particle. We chose the indexes i, j, k to denote particle number, particle type, and cell-id, respectively. With this notation, $\xi_{i,j,k}$ is the thermal fluctuation felt by each particle and $F_C(y_{i,j,k})$ is the net pairwise force acting on it. In particular, Newman used the following Morse-like elastic potential ($V(d)$ where d is the inter-particle distance) to enforce adhesion and volume exclusion (Fig 1B and Eq. 2) [16,18].

$$V(d) = u_0 e^{2\rho\left(1-\frac{d^2}{d_{eq}^2}\right)} - \alpha u_0 e^{\rho\left(1-\frac{d^2}{d_{eq}^2}\right)} \qquad (2)$$

In Eq. (2), u_0 is the potential's well depth, ρ and α are scaling and shifting factors, and d_{eq} is the equilibrium distance between particles. To limit the computational cost, these potentials focus on short-range interactions and cut off at 2.5 times the equilibrium distance. To assign the forces in Eq. (1) based on cell-level rheology, Newman assumed N_p densely packed particles connected by springs and recovered the following relationships[16]

$$d_{eq} = 2R_{cell}\left(\frac{p_d}{N_p}\right)^{\frac{1}{3}} \qquad (3a)$$

$$\eta = \frac{\eta_0}{N_p} \qquad (3b)$$

$$\kappa = \kappa_0 N_p^{-\frac{1}{3}}\left(1 - \lambda N_p^{-\frac{1}{3}}\right) \qquad (3c)$$

$$u_0 = \kappa \frac{d_{eq}^2}{(8\rho^2)} \qquad (3d)$$

In Eqs. (3a-3d), p_d is the sphere close packing density, R_{cell}, is the radius of the cell, κ_0 and η_0 are the stiffness and viscosity of the cytoplasm, respectively. λ is a tuning coefficient which is an adjustment to take care of the departure from a cubic lattice arrangement used to derive the elastic modulus of the network [16]. The list of parameters and their values are listed in Table 1. We have inherited some of the basic parameter values, related to the functional form of the potential, such as the scaling factor,

shifting factor, tuning coefficient from an earlier SEM model [18]. The model is calibrated on a particular cell type and ideally it should be applied on that same cell type and similar experimental conditions for quantitative match with experiments.

Table 1: Parameters used in SEM^2

Parameter	Description	Value
R_{cell}	Cell radius	10 µm
m_{cell}	Cell mass	3.1 ng
N_p	Total number of particles in a cell	1000
p_d	Sphere close packing density	0.74
ρ	Scaling factor	2
α	Shifting factor	2
κ_0	Cell stiffness	1.2×10^{-2} N m^{-1}
η_0	Cell viscosity	5×10^{-2} N s m^{-1}
λ	Tuning coefficient	0.75
m_p	Mass of each particle	3.1×10^{-3} ng

Comment 1.4. The authors rigorously calibrate their model to experimental results, which is commendable. However, different cell types are used for calibration. Realistically, mechanical properties differ between cell types and could potentially vary over time, e.g. as a cell switches from stationary to motile states. The authors should comment on this in the discussion.

Response 1.4
The model parameters are indeed dependent on a particular cell type and ideally should be executed on experiments carried out on cells of the same cell type. We also acknowledge the variation of a cell's mechanical properties at different time points, such as the stationary and motile states of a cell. We acknowledge this limitation of our model and explicitly mention it in the Discussion.

Modification 1.4
In Discussion, after the sentence:

"BIOPOINT's validation across multiple experimental conditions supports its application for investigating nuclear mechanics in morphogenesis."

we have added the following text

"We calibrated parameters on a particular cell type and cross-validated it across different cell types depending on the data we found in the literature. While the semi-quantitative agreements (Fig. 3) indicate robustness of our emergent property predictions, high-accuracy simulations may need the model to be calibrated and validated against data collected on the same cell type, especially as mechanical properties may vary across cell types, but also in the same cell type in different situations [40–42]. A discussion on how the parameter values are expected to change in different cell types is provided in SI."

Comment 1.5. What interaction potential did the authors choose for interactions between nucleus and cytoplasm particles? How does the model ensure that the nucleus particles stay surrounded by cytoplasm particles?

Response 1.5

We introduced text in the manuscript to better describe this important contribution. The potential between nucleus and cytoplasm has the same functional form as that between nuclear particles or between cytoplasmic particles. The strength of the potential (u0) is ~ 1.5 times that between cytoplasmic particles, modeled through the parameter, k12, which is also optimized. Now, we have added Fig. S5 where we plot the inter-type (cytoplasm-nucleus) potential along with the cytoplasm-cytoplasm and nucleus-nucleus potential. Since the inter-type potential is stronger than the cytoplasmic potential, the cytoplasmic particles have an affinity towards the nuclear particles, ensuring that they surround them. Additionally, the biasing force on the nucleus towards the cell center also ensures that the nucleus is surrounded by cytoplasmic particles.

Modification 1.5
We have added the following sentence in Methods in the subsection, titled "Preparation of multi-particle nucleus":
"The biasing force on the nucleus towards cell center, along with the superior cytoplasmic-nuclear adhesion compared to the cytoplasmic-cytoplasmic adhesion (Fig. S5), ensures that it is always surrounded by cytoplasmic particles."

We have added Fig. S5 in the Supplementary Information to plot the inter-type (cytoplasm-nucleus) potential, along with the cytoplasm-cytoplasm and nucleus-nucleus potential.

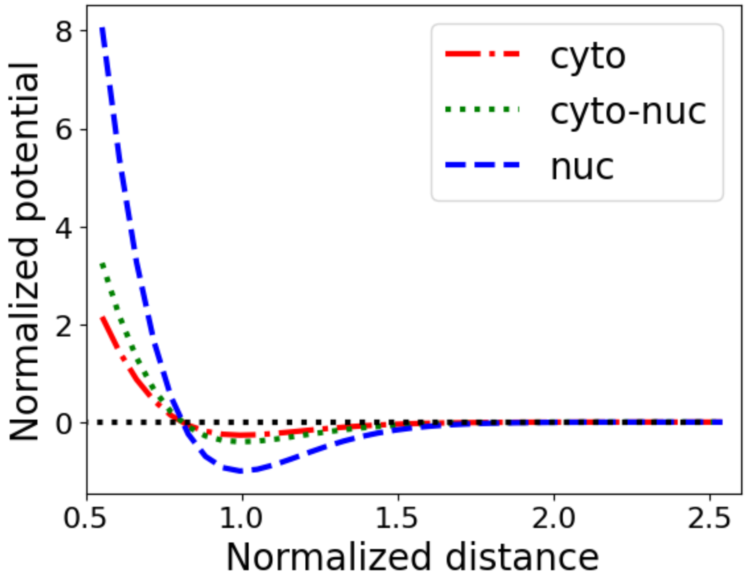

Comment 1.6. Regarding Figure 3G: The line connecting the dots for "Exp:hMSC" does not look correct. It seems to move backwards along the x-axis. Something similar occurs in Figure S3A in the supplementary material.

Response 1.6

Thanks for pointing this out. This discrepancy arose because of a typo in the plotting routine which is now fixed. All code is available in our GitHub repository.

Modification 1.6

Fig. 3G has been modified.

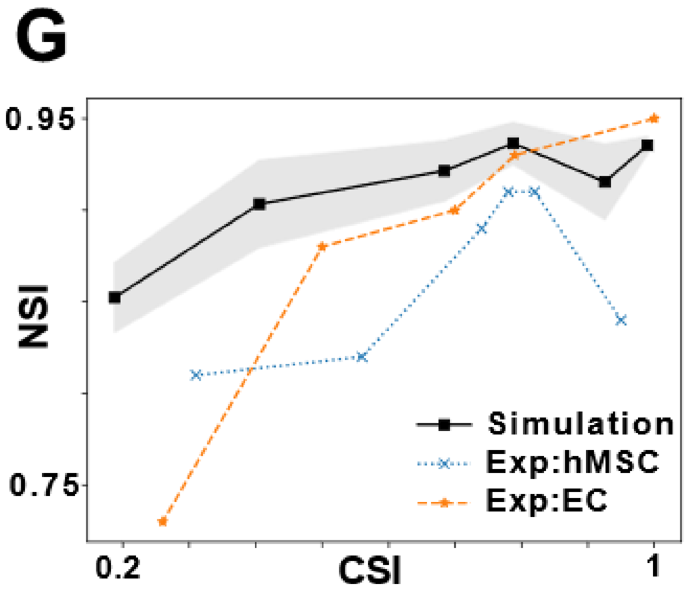

Comment 1.7. The nuclear deformation in the experimental images of Figure 4A looks very extreme compared to the simulation in Figure 4C. Yet the bars in the quantification in Figure 4B show the opposite: The simulation appears to have a more extreme NSI than the experiment. Can the authors explain why?

Response 1.7

We again thank the reviewer for taking note of this discrepancy. This discrepancy arose because the convex hull algorithm to create a boundary around the 2D coordinates, was not taking into account the tapering at the centre.

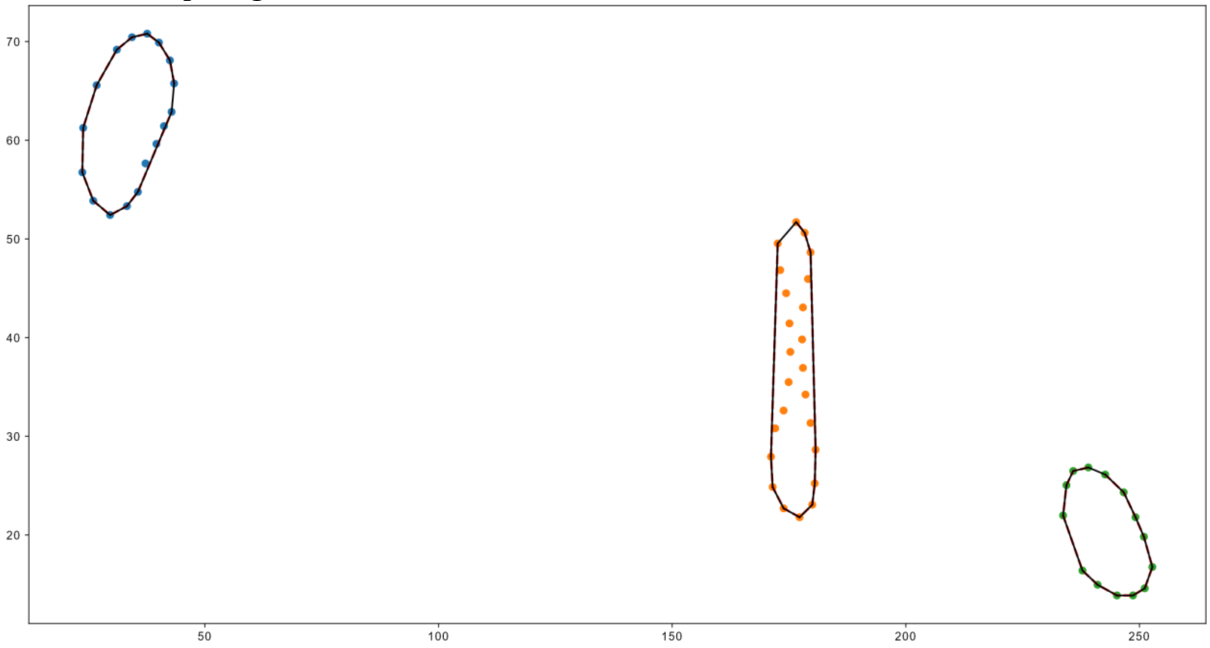

We have now corrected for this using alpha shape instead of convex hull.

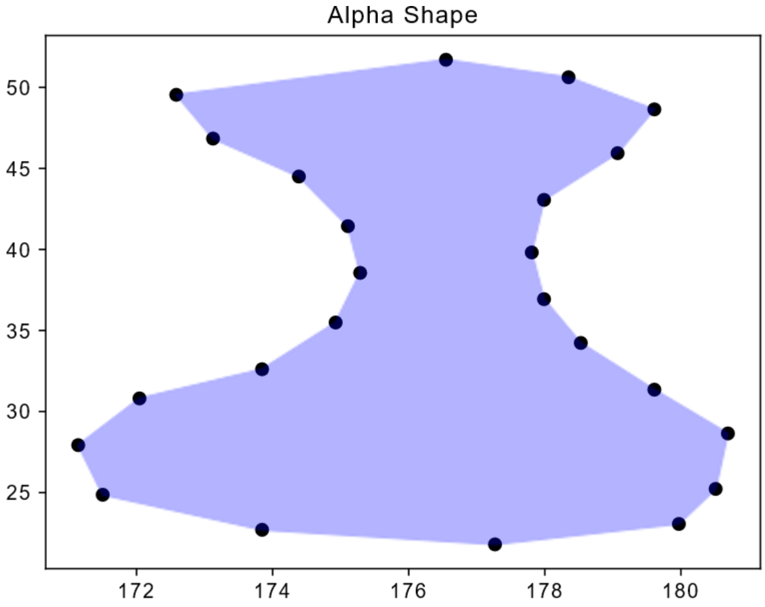

Modification 1.7
The modified Fig. 4B is as follows:

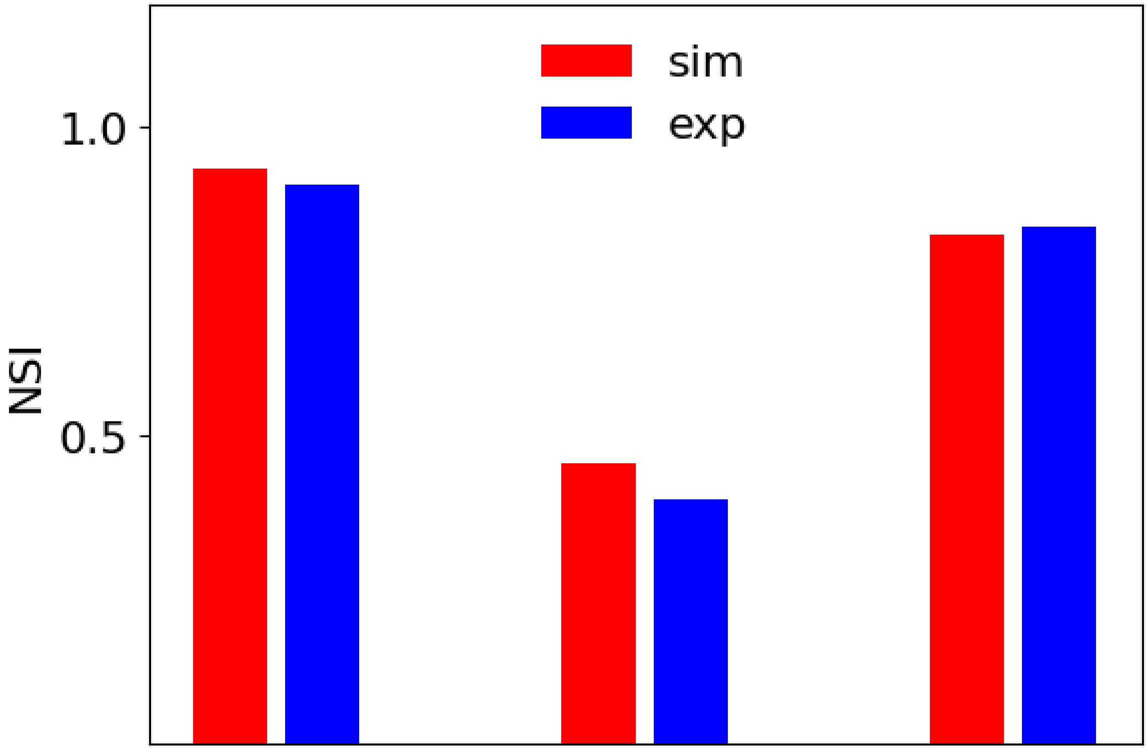

The following text has been added in the Methods section:
"For extremely tapering shapes, such as the experimental cell shape inside the constriction, we use alpha shape method instead of convex hull method as it gives more accurate estimation of nuclear shape."

Comment 1.8. Did I understand right that all cytoplasmic particles can adhere to the ECM and thus there is not a special "membrane" particle? Does this mean that the extent of cell spreading is mediated by the balance between cytoplasm-ECM adhesion and cytoplasm-cytoplasm adhesion? Or will all cells eventually maximally spread once equilibrium is reached?

Response 1.8

Yes, there are no dedicated membrane particles. Unless one wants to introduce an additional, phase-separated, particle type to track the membrane, it's difficult to have proper SEM and a membrane representation. This is why vertex modeling approaches (or hybrid ones like the one discussed in Comment 1.1) elect to track the membrane only. Instead, we followed the SEM++ convention from Petros Koumoutsakos' group: each particle carries a cell_id and the outermost particles of each cell are recognized by the code as membrane particles. In this framework, the extent of cell spreading is mediated by the balance between the cytoplasm-ECM adhesion and cytoplasm-cytoplasm adhesion which is handled by the parameters corresponding to the well-depth of the respective potentials -- ε for the LJ potential between cytoplasm-ECM and u0(1-1) for the Morse potential between cytoplasm-cytoplasm. If ε is much higher compared to u0(1-1), the cell will maximally spread. However, the nucleus will not spread so much as there is no potential between nuclear and ECM particles. We have now provided a better explanation of the balance between the well depths of the LJ and Morse potentials for cytoplasm-ECM and cytoplasm-cytoplasm, respectively.

Modification 1.8

We have added the following sentence in Methods, in the subsection titled "Cell spreading":

"The degree of cell spreading on ECM substrate depends on the parameters corresponding to the well-depth of the respective potentials -- ε for the LJ potential between cytoplasm-ECM and $u_0(1-1)$ for the Morse potential between cytoplasm-cytoplasm."

Comment 1.9. What is the lowest number of nuclear or cytosolic particles that can still reproduce the experimental data? This would be interesting to know, given that computational cost scales with number of particles. In a similar vein, why did the authors choose 100 nucleus particles?

Response 1.9

A minimum of 100 particles per cell has been recommended in the seminal SEM paper (Sandersius et al. 2005) to reproduce realistic cell shapes. Our assumption from there is that we must allocate 100 particles for the nucleus, which is ~10% of the cell volume, resulting in a 100/900 nuclear/cytoplasmic particle combination for standard conditions. A higher number of particles would increase computational cost due to more particle interactions and also need a lower timestep, thus increasing computational time significantly, which would make the optimization procedure or long-range simulations difficult. As an example, we prepared a cell with twice the nuclear size, i.e. 200/800 nuclear/cytoplasmic particle combination. The results of cell spreading on this cell is provided in Supplementary Figure S6.

Modification 1.9

We have added the following sentence in Methods, in the subsection titled "Cell spreading simulations on ECM patterns":

"We chose 100/900 nuclear/cytoplasmic particles in order to have the minimum number of nuclear particles to reproduce emergent nuclear shapes [16], but conducted additional simulations with 200 nuclear particles representing the nucleus (see SI, Fig. S6)."

We have added the following text in the SI:

"In order to investigate the effect of nuclear size, we have designed a cell with twice the nuclear size, i.e. 200 nuclear particles and 800 cytoplasmic particles. We have performed cell spreading on the various ECM patterns that were used in Fig. 3. We repeated the analyses of cell shape and nuclear shape for this cell with larger nucleus. We didn't observe that the nucleus deforms much more than the smaller nucleus comprising 100 particles. This suggests that in the simulation scenarios studied here the potentials governing the nuclear, cytoplasmic and ECM particles and the interplay between them plays a bigger role in nuclear deformation than nuclear size."

We have added supplementary figure S6 to show the effect of increasing the number of particles in the nucleus to 200. (see the next page)

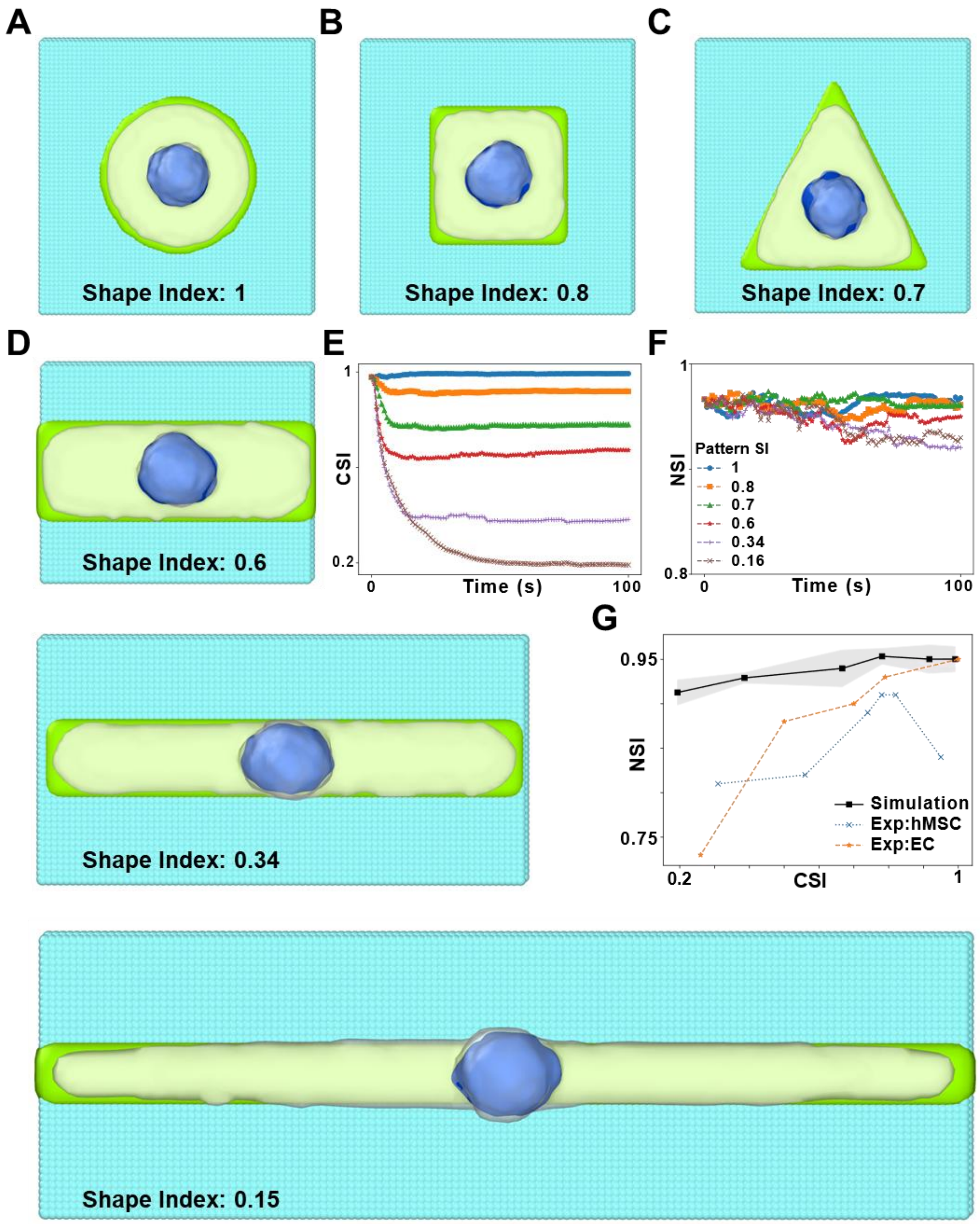

Figure S6. Evolution of shape in the cell with 20% nucleus -- 200/800 nuclear/cytoplasmic particles. Simulations of cell spreading on circular (A), square (B), and triangular (C) ECM patterns, where the cell takes up the shape of the pattern. The cell membrane is depicted in white, the nucleus in blue, the ECM surface in green, and surrounding glass particles in cyan. D) Cell spreading on three rectangular patterns with similar area but different shape index. E) Cell shape index (CSI) versus time as the cell spreads on the various ECM patterns. F) Nuclear shape index (NSI) versus time during cell spreading on the various ECM patterns (legends of E and F are the same). G) Plot of NSI versus CSI for our simulations and experimental results from the literature: EC [29] and hMSC [30].

Comment 1.10. For Figure 2C and 2D, the authors do an uncertainty quantification (UQ). I had some trouble understanding what these panels show, and would appreciate more explanation.
I understand from the methods that the authors varied parameter values and repeated the simulation using a Python package. Is 2C showing summary statistics of predictions assuming the parameters vary around a given starting value? How much do the starting values affect the outcome of the UQ? Is this what 2D is showing?

Response 1.10
We performed UQ with a 4th-order polynomial chaos expansion using independent Normal priors on six parameters (cytoplasm stiffness, cytoplasm viscosity, nucleus stiffness, nucleus viscosity, λ, k12). Each prior was centered at the nominal value in Table 3 (our "starting value") with σ = 0.1·μ. Figure 2C plots the PCE-predicted mean (μ) and standard deviation (σ) of the AFM force–time curve—i.e., propagation of parameter uncertainty around those starting values. Figure 2D shows first-order Sobol indices $S_i(t)$ computed from the PCE coefficients; these quantify each parameter's contribution to output variance over time and do not depict sensitivity to the absolute starting values. In our case, nuclear stiffness and k12 dominate the variance of the force–time curve, while λ contributes minimally, with cytoplasmic terms secondary.

Modification 1.10
We added the following info to the caption of Figure 2:
"2C: PCE μ±σ band of the indentation force vs time under Normal priors (σ = 0.1·μ) centered at Table 2 nominal values for six parameters. 2D: First-order Sobol indices $S_i(t)$ from the same PCE, showing variance contributions of each parameter over time. (Higher S_i ⇒ larger share of output variance.)"

We have added the following sentence in Methods, in the subsection titled "Uncertainty Quantification":

We used EasyVVUQ [46,47] with Chaospy [48] to build a 4th-order polynomial chaos expansion (PCE) for the indentation force–time output. Priors were independent Normal distributions centered at the nominal (Table 2) parameter values with σ = 0.1·μ. Varied parameters: cytoplasm stiffness, cytoplasm viscosity, nucleus stiffness, nucleus viscosity, λ (tuning coefficient), and k_{12} (relative nuc–cyto interaction strength). From the PCE coefficients we computed moments (μ, σ) and first-order Sobol indices $S_i(t)$ for the time-resolved force.

Comment 1.11. The authors show some interesting data in the supplementary material. For example Figure S4 is quite nice, and in this reviewer's opinion should be showcased in the main manuscript, perhaps in Figure 2.

Response 1.11
We thank the reviewer for appreciating the data in the supplementary material. We have brought Figure S4 to the main manuscript (Fig. 4). We have also modified the text to embed this figure in the main manuscript.

Modification 1.11
In the subsection, titled, "Validation of BIOPOINT via Cell Spreading on ECM Patterns", the following text has been modified from
"Additionally, the stresses in spreading are much smaller than those in indentation, as can be seen from the spatial distribution of stresses and spatial binning plots (Fig. S4), which might non-linearly affect nuclear response."

To
"Additionally, the stresses in spreading are much smaller than those in indentation, as can be seen from the spatial distribution of stresses and spatial binning plots (Fig. 4), which might non-linearly affect nuclear response. The visualization of nuclear stress in the spreading (Fig. 4A) and indentation (Fig. 4B) simulations show that there are considerably higher stresses in the nucleus during indentation. This can be further corroborated from the plot of average stress per particle in spatial bins along the x axis (Fig. 4C). The absolute values of stresses are much more in the indented nucleus as compared to the nucleus in the spread-out cell."

The new Fig. 4 is below for convenience.

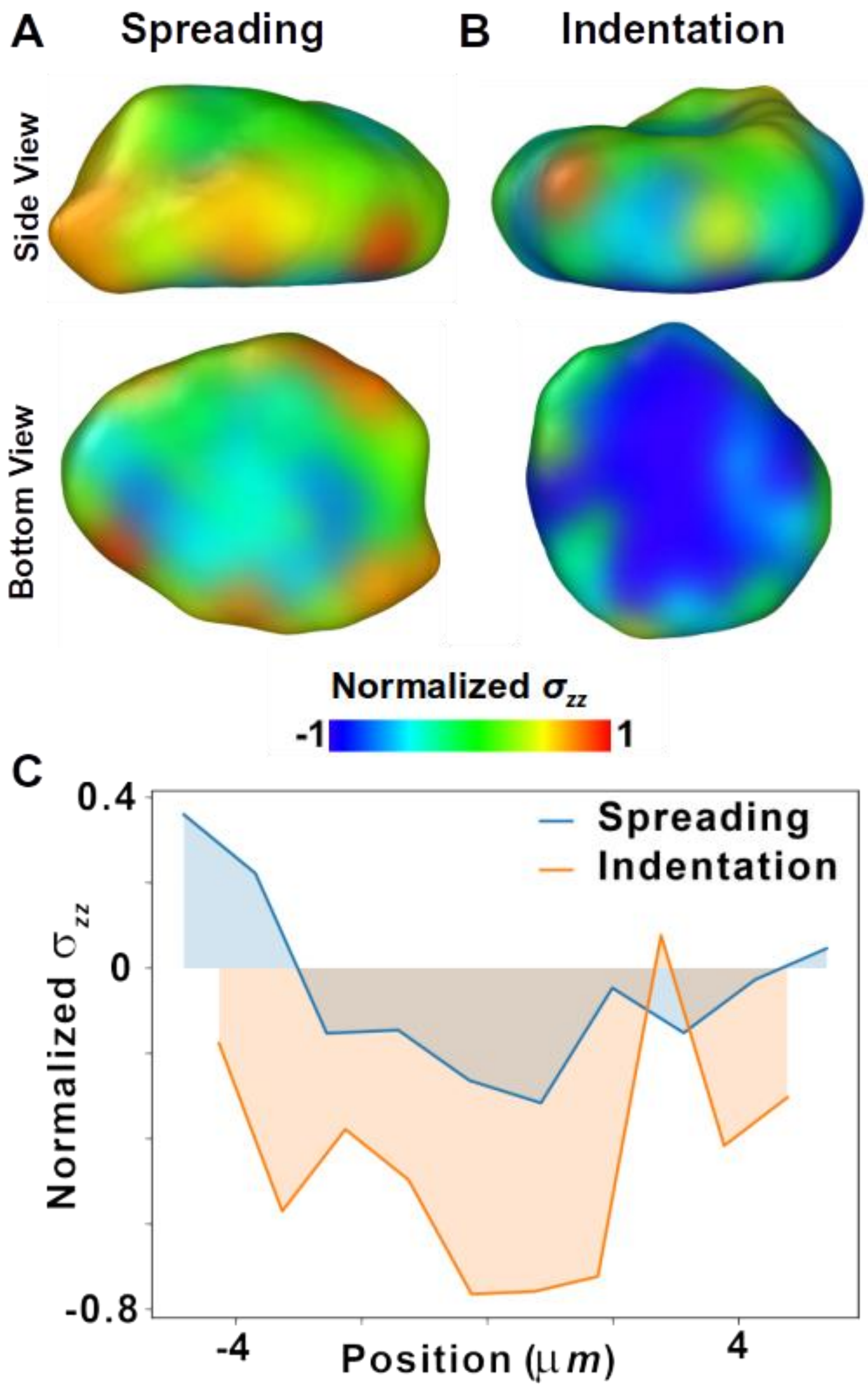

Figure 5. Distribution of nuclear stress during indentation and spreading simulations. Side view and the bottom surface view of A) a nucleus of a cell spread on ECM substrate and B) an indented nucleus. The nucleus has been color coded with normal component of the per-particle stress tensor (σzz). C) Plot of normalized σzz versus position along one-dimensional spatial bins along the x axis.

Comment 1.12. About supplementary Figure S2, where the authors show the spatial stress distribution at the bottom surface of a cell adherent to the ECM. Why is there a tensile/positive stress in the boundary and a compressive/negative stress within the cell? Do the authors have an explanation of how this stress distribution emerges?

Response 1.12

This is an excellent question. Please note that we haven't explicitly modelled membrane particles. As discussed earlier, membrane particles are recognized as the outer-most particles of every cell (following the convention introduced by the Koumoutsakos group in their SEM++ paper, Milde et al. 2014) However, as an emergent property of the simulations, we find that there is a greater presence of positive/tensile stress on the peripheral particles and negative/compressive stress on the particles inside the cell. This is probably because the particles at the periphery are more stretched (from their equilibrium distances) than the ones in the interior, which are at relatively shorter distances – in good agreement with our observation in growing 3D tissues in our previous publication (Chattaraj et al., APL Bioengineering, 2023).

Modification 1.12

We have modified the following text in the subsection titled "Validation of BIOPOINT via Cell Spreading on ECM Patterns" from:

"The stress distribution in the spread-out cell can be visualized from the computation of per-particle stress (Fig. S2)."

to

"The stress distribution in the spread-out cell can be visualized from the computation of per-particle stress (Fig. S2). Please note that we haven't explicitly modelled membrane particles. However, as an emergent property of the simulations, we find that there is a greater presence of positive/tensile stress on the peripheral particles and negative/compressive stress on the particles inside the cell."

We have also added the following text in SI"

"There is a greater presence of positive/tensile stress on the peripheral particles and negative/compressive stress on the particles inside the cell. This is probably because the particles at the periphery are more stretched (from their equilibrium distances) than the ones in the interior, which are at relatively shorter distances. We have discussed this phenomenon in detail, in 3D in our previous publication (Chattaraj et al., APL Bioengineering, 2023)."

Comment 1.13. For Figure 2, the authors state: "A BIOPOINT cell was spread on an ECM layer, with the nucleus positioned near the top surface." From the wording it's unclear if this behavior is an emergent property of the simulation or if the authors encoded this configuration.

Response 1.13
We better clarified this aspect in the text. The phenomenon emerges as a result of the fact that there is an attractive potential between the cytoplasmic and ECM particles but not between nuclear and ECM particles. The cytoplasmic particles spread over the ECM particles with those above the nuclear particles sliding past them. The degree of the spreading depends on the balance between the potential strength of cytoplasmic-ECM, cytoplasmic-nuclear and cytoplasmic-cytoplasmic interactions.

Modification 1.13
We have added the following text after the above-mentioned sentence:
"This is a result of the fact that there is interaction between the cytoplasmic and ECM particles but not between nuclear and ECM particles."

Comment 1.14. The authors state that BIOPOINT "optimizes cell mechanics" (last paragraph before Figure 2 is shown). What do the authors mean by "optimize"? Perhaps they could reword for clarity.

Response 1.14
We have reworded this sentence for better clarity. Thanks.

Modification 1.14
We modified the sentence:
“Thus, BIOPOINT accurately reproduces experimental force-time curves, identifies dominant mechanical parameters, and optimizes mechanical parameters to match experimental results, ensuring a biophysically accurate, experimentally validated framework.”

Comment 1.15. About the parameter uncertainty quantification: To what extent are the values of the parameters uniquely identifiable?

Response 1.15
We have varied the parameters within a physiologically relevant range based on previous publications. Since the indentation experiments on a stretched-out cell on ECM mostly involves deforming the nucleus, we assumed that the nuclear stiffness and viscosities might impact the force-time curve the most. This helped us to guess the initial parameter values. With the uncertainty quantification, we were able to find the sensitivity of the force-time curve to individual parameters. Furthermore, we performed the optimization with a reasonable initial guess of parameter values and obtained parameter values which give us a good fit to the experimental force-time curve. Thus, the parameters were obtained from the optimization and not the UQ. Like with most highly parametrized model, we acknowledge that the parameter values might not be unique and there might be other combinations which also give us a good match with the experimental data. However, the fact that we can simulate very different scenarios in the following figures and obtain quantitative or qualitative agreement on emergent trends, suggests that our parameter set is not overfitted to the indentation datasets. We modified our manuscript to better discuss this aspect.

Modification 1.15
In the last paragraph of the "Calibration" section, we have modified the following text

"Upon convergence (Fig. 2E), BIOPOINT learned parameter values that successfully matched experimental force-time curves, confirming that the model's elastic and viscous properties aligned with real cell behavior (Fig. 2F). Thus, BIOPOINT accurately reproduces experimental force-time curves,..."

to the following text

"Upon convergence (Fig. 2E), BIOPOINT learned parameter values that successfully matched experimental force-time curves, confirming that the model's elastic and viscous properties aligned with real cell behavior (Fig. 2F). While we acknowledge that this might not be a unique set of parameter values which give us a good match with the experimental force-time curve, since we have probed a physiologically relevant parameter range and the initial guesses were obtained based on the physical meaning of parameter values, we assume that we determine properties which are close to reality. Thus, BIOPOINT accurately reproduces experimental force-time curves,..."

Comment 1.16. Regarding Figure 3F: It would improve figure legibility if the legend was moved outside the plot area and if the points were connected by lines.

Response 1.16
We thank the reviewer for the suggestion. We have made the necessary modification.

Modification 1.16
Fig. 3E and 3F have a common legend. Fig. 3E has been modified. The modified Fig. 3 is as follows.

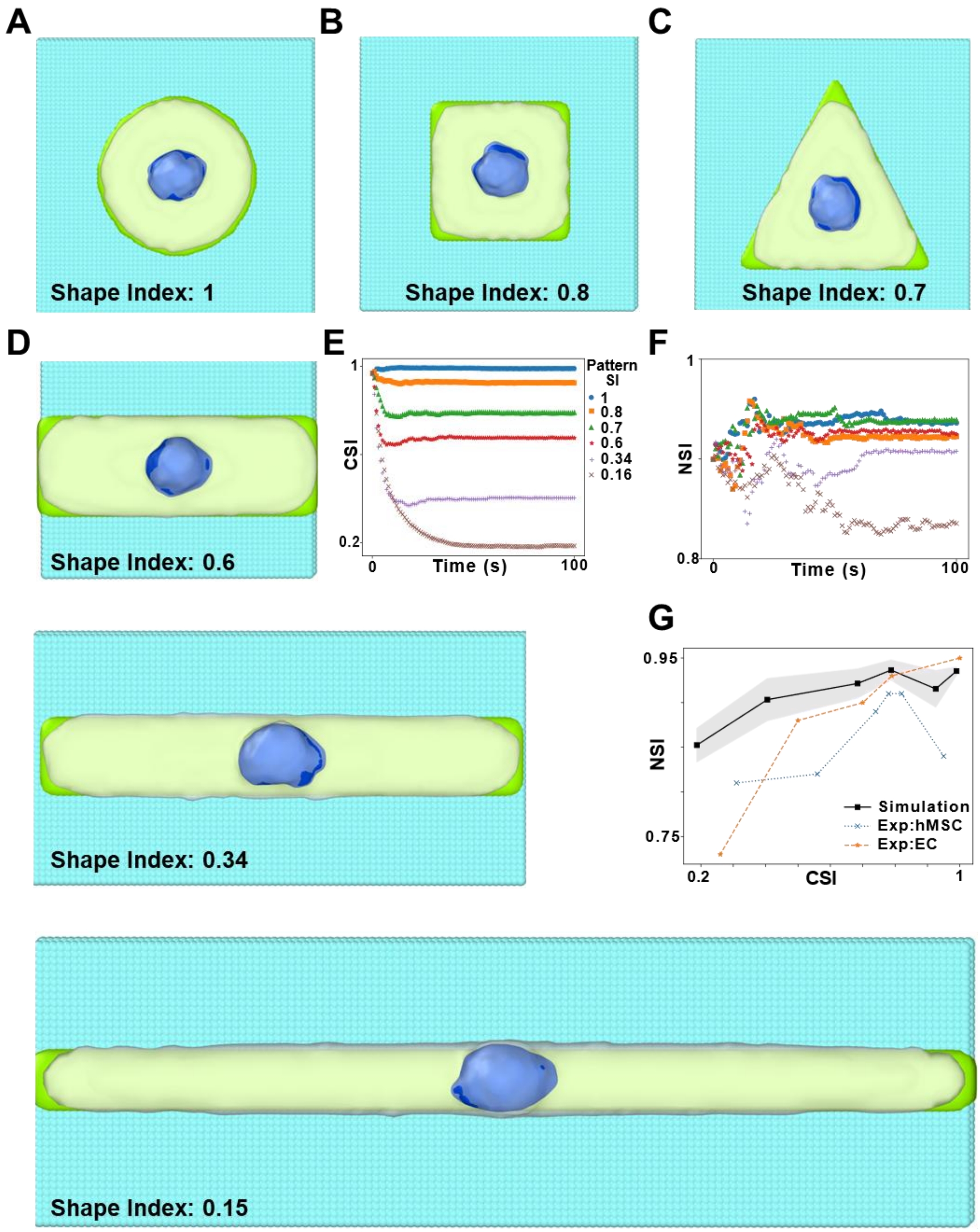

Reviewer #2

Comment 2.0

The authors have presented a method of performing simulations of single cells with a particle representation for both the nucleus and the cytoplasm. The advance here is that while in previous work, the nucleus was represented by a single rigid particle, here, the nucleus is represented by a number of smaller particles that experience a potential interaction with each other. The expectation is that nuclear particles, experiencing certain potential-based interactions with each other, will naturally cluster and phase separate from the cytoplasmic particles. Hence the model is expected to behave similarly to a 2-phase model.

The model presented here depends on multiple parameters including single particle stiffness parameters and particle-to-particle interaction strengths. The utility of the model is that once these parameters are trained on data, they can be used on future numerical experiments for the same cell type. I believe this method could be useful to reserachers that have access to single cell experiments and are therefore able to calibrate the model. After that, the calibrated model could be used as a predictive tool for future experiments on the same cell type.

*The strength of the manuscript is that researchers may indeed find the model useful and implementable in their own labs. The weakness of the manuscript is that although it presents a useful tool, it does not give insight to biological phenomena or biophysics. The majority of the comments I have about the paper is regarding its clarity when it comes to explaining and presenting the model, as well as asking for more complete justification to the modeling assumptions and methods. The points with ** are likely the more important points to address.*

Response 2.0
We appreciate the reviewer's summary of our work and the provided inputs. We have addressed the comments and made necessary changes to the manuscript and SI as can be found below.

Modification 2.0
No modifications.

Comment 2.1. I want to stress (from what I understand) that this model may be limited to the use case I mentioned earlier, i.e. it is a numerical model that requires calibration before use; and future applications of the model must be on the same cell type and similar experimental conditions as when it was calibrated. The authors should mention this use case and limitation.

Response 2.1
We agree with the reviewer that the best way to use our model - like any highly parametrized computational model - is to fit its parameters to the specific cell and experiment under consideration. With that said, SEM models are particularly noteworthy for their ability to have cell shape as an emergent property and we were relieved that BIO-POINT exhibited emergent properties that were well controlled by biophysically-sound parameters. For example, the degree of nuclear deformation on the ECM pattern was controlled by the nuclear to cytoplasm potential strength, which let us model different cell types by refitting a single parameter with a clear biophysical meaning.

In other words, BIO-POINT qualitatively reproduces ranges of relevant biological observations but requires parameter calibration if one is interested in quantitatively modeling a given cell type or experiment. We have calibrated the model parameters with the force-time curve from single cell indentation experiments as a reference. Ideally, one should apply the model on the same cell type and similar experimental conditions. We have now explicitly mentioned this use case as a limitation.

Modification 2.1
In Discussion, after the sentence:

"BIOPOINT's validation across multiple experimental conditions supports its application for investigating nuclear mechanics in morphogenesis."

we have added the following text

"We calibrated parameters on a particular cell type and cross-validated it across different cell types depending on the data we found in the literature. While the semi-quantitative agreements (Fig. 3) indicate robustness of our emergent property predictions, high-accuracy simulations may need the model to be calibrated and validated against data collected on the same cell type, especially as mechanical properties may vary across cell types, but also in the same cell type in different situations [40–42]. A discussion on how the parameter values are expected to change in different cell types is provided in SI."

Comment 2.2. The paper would benefit in general from the Methods section being merged with the main text, or for some portion of the Methods to be included in the main text. For example, the parameter \kappa_nuc and \kappa_0 appear without explanation in the main text other than to say they are a "stiffness potential". Equations like (4) and (5) could be moved to the main text. The parameter tables should also be moved to the main text. The paper is about your method, so all the parameters should appear in the main part of the paper.

Response 2.2
We really appreciate this feedback from the reviewer. We have now added a subsection, "Theory of SEM", at the end of Introduction and moved parts of the "Methods" section here. Parameter \kappa_nuc and \kappa_0 are better explained here. We have moved Equations (4) and (5) and the parameters table to this subsection.

Modification 2.2
In Results, we have added a subsection, titled "Theory of SEM". We have explained all the SEM parameters here and the additional nucleus and ECM-related parameters in the next subsection. We also listed the SEM and additional parameters in Table 1 and Table 2, in the main text. Please see the modification with respect to the editor's comment at the beginning of this document.
Please note the following texts:
" κ_0 and η_0 are the stiffness and viscosity of the cytoplasm, respectively."
"κ_{nuc} and η_{nuc} are respectively the stiffness and viscosity of the nucleus."

Comment 2.3. The "stiffness potentials" all need to be explained with a functional form or physical descriptor (is it Young's modulus? Is it elastic?), something beyond saying it is parameter found in LAMMPS.

Response 2.3
We acknowledge the reviewer's concern here. We have provided the functional forms of the potentials in Eqs. 2 and 5 in the Theory of SEM section for cellular and cell-ECM interactions, respectively.

Modification 2.3
We have provided the following equations and text in the "Theory of SEM" subsection:

"In particular, Newman used the following Morse-like, elastic potential ($V(d)$ where d is the inter-particle distance) to enforce adhesion and volume exclusion (Fig 1B and Eq. 2) [16,18].

$$V(d) = u_0 e^{2\rho\left(1-\frac{d^2}{d_{eq}^2}\right)} - \alpha u_0 e^{\rho\left(1-\frac{d^2}{d_{eq}^2}\right)} \quad (2)$$

In Eq. (2), u_0 is the potential's well depth, ρ and α are scaling and shifting factors, and d_{eq} is the equilibrium distance between particles. To limit the computational cost, these potentials focus on short-range interactions and cut off at 2.5 times the equilibrium distance."

For cell-ECM interactions, we added the following text in the subsection, titled "BIOPOINT uses heterogeneous discrete particle types to model the cell nucleus and ECM":

"The attraction between cytoplasmic and ECM particles is modeled by a Lennard-Jones (12-6) potential:

$$V(d) = 4\varepsilon\left[\left(\frac{\sigma}{d}\right)^{12} - \left(\frac{\sigma}{d}\right)^{6}\right] \quad d < d_c \quad (5)$$

Where d is the distance between the cytoplasmic and ECM particles, σ is the zero-crossing distance, ε is the potential well-depth, and d_c is the cutoff distance."

*Comment 2.4a**. Similar to 3, all parameters all need to be defined and explained in the main text. Even in the Methods section, explanations are missing. For example, in the text prior to Eq. (6), the parameters are mentioned only by name without explanation. What is tuning coefficient? What is u_0 in Eq. (6)? Eq. (6) itself is not clear, what is u_0(1-2)?*

Response 2.4a
We thank the reviewer for giving us the opportunity to improve the "Methods" section. We have addressed these concerns by providing explicit descriptions of all parameters in the Theory of SEM

Modification 2.4a
The following text in subsection, titled "BIOPOINT uses heterogeneous discrete particle types to model the cell nucleus and ECM." provides an explanation for the above-mentioned parameters:
"The relative stiffness of cytoplasmic-nuclear interaction, in comparison to cytoplasmic stiffness, is represented by the parameter, k_{12}.

$$k_{12} = \frac{u_0(1-2)}{u_0(1-1)} \quad (4)$$

$u_0(1-2)$ and $u_0(1-1)$ represent potential well depth for the cytoplasm-ECM and cytoplasm-cytoplasm particle interactions, respectively, 1 and 2 representing particle type, as denoted by the index j in Eq. (1).

Additionally, the following text can be found in the subsection "Thoery of SEM"

λ is a tuning coefficient which is an adjustment to take care of the departure from a cubic lattice arrangement used to derive the elastic modulus of the network [16]. We have inherited some of the parameter values, related to the functional form of the potential, such as the scaling factor, shifting factor, tuning coefficient from an earlier SEM model [18]."

*Comment 2.4b**. Viscosity is not explained in the main text, how is viscosity simulated between particles? Viscosity, scaling factor, shifting factor, etc all appear in the Methods, they all need explanation.*

Response 2.4b
We have provided a better description of viscosity and other parameters in our Theory of SEM and updated Methods section.

Modification 2.4b
The following text has been provided in the Theory of SEM subsection:
"In Eqs. (3a-3d), p_d is the sphere close packing density, R_{cell}, is the radius of the cell, κ_0 and η_0 are the stiffness and viscosity of the cytoplasm, respectively. The viscous force acting on each particle is given in the left-hand side (LHS) of Eq. (1)."

Comment 2.5. How the per-particle stress is determined needs to be explained (other than it comes from LAMMPS). The explanation of how the stress is projected onto the nuclear surface should explained better.

Response 2.5
A better description of per-particle stress computation is provided in the updated Methods section.

Modification 2.5
The following text has been included in the Methods section:

"***Particle stress*:** The per-particle stress has been determined utilizing the "compute stress/atom" functionality of LAMMPS. This yields per-particle virial stress, which has the units of energy and can be converted to stress units if divided by the particle volume. We compute the mean volumetric stress of each particle as, $\sigma_m = \frac{\sigma_{xx}+\sigma_{yy}+\sigma_{zz}}{3}$. In order to distinctly visualize the distribution of stresses across the subcellular material, we have normalized the stresses with maximum and minimum values of a range within which stresses of the majority of particles lie. In order to project particle stress onto the surface, we used OVITO [44], where existing attributes of the input particles located at the surface are copied over to the vertices of the constructed surface mesh."

*Comment 2.6**. How is the membrane defined and evolved?*

Response 2.6
We followed the SEM++ convention from Petros Koumoutsakos' group: each particle carries a cell_id and the outermost particles of each cell are recognized by the algorithm as membrane particle. There is no particle type, which explicitly describes the membrane. The peripheral particles are considered to comprise the membrane. We find that the stress distribution in these peripheral (or membrane) particles is different from the rest of the cell, which is an emergent property of our simulations.

Modification 2.6
We have modified the following text in the subsection titled "Validation of BIOPOINT via Cell Spreading on ECM Patterns" from:

"The stress distribution in the spread-out cell can be visualized from the computation of per-particle stress (Fig. S2)."
to
"The stress distribution in the spread-out cell can be visualized from the computation of per-particle stress (Fig. S2). Please note that we haven't explicitly modelled membrane particles. However, as an emergent property of the simulations, we find that there is a greater presence of positive/tensile stress on the peripheral particles and negative/compressive stress on the particles inside the cell."

We have also added the following text in SI"
"There is a greater presence of positive/tensile stress on the peripheral particles and negative/compressive stress on the particles inside the cell. This is probably because the particles at the periphery are more stretched (from their equilibrium distances) than the ones in the interior, which are at relatively shorter distances. We have discussed this phenomenon in detail, in 3D in our previous publication (Chattaraj et al., APL Bioengineering, 2023)."

*Comment 2.7**. The fit or calibration of the adhesion potential between ECM particles and cytoplasmic particles are never presented (or I just haven't found it). There are 3 parameters associated with Lennard-Jones, how were these 3 parameters fitted in your paper? Their values are also not given in the Methods.*

Response 2.7
The parameters of the Lennard-Jones potential have been obtained from cell spreading experiments where the cell height has been matched to the cell height of the indentation experiments of Hobson et al. We have added a subsection in the SI where we describe how we have obtained the LJ parameters.

Modification 2.7
After the following text, "d is the distance between the cytoplasmic and ECM particles, σ is the zero-crossing distance, ε is the potential well-depth, and d_c is the cutoff distance."

We added the following text:
"Details about the determination of LJ potential parameters is provided in SI."

Additionally, we added the following text to SI.

Determining the LJ potential parameters

The ECM particles act like a planar wall that attracts the cytoplasmic particles, resulting in cell spreading. In the scope of this work, there is no interaction between ECM particles. The LJ potential between cytoplasmic and ECM particles has been provided in Eq. 5 of the main manuscript. The attraction between cytoplasmic and ECM particles is modeled by a Lennard-Jones (12-6) potential:

$$V(d) = 4\varepsilon\left[\left(\frac{\sigma}{d}\right)^{12} - \left(\frac{\sigma}{d}\right)^{6}\right] \quad d < d_c \qquad (5)$$

d is the distance between the cytoplasmic and ECM particles, σ is the zero-crossing distance, ε is the potential well-depth, and d_c is the cutoff distance. The gap between the planar ECM particles and the cytoplasmic particles is fixed for visualization purposes, such that σ = 5µm. This value can be tuned to adjust the gap between the ECM and the cell. The cut-off distance, d_c, is set as the sum of σ and the radius of the cell, so that there is interaction between the particles in the bottom hemisphere of the cell and the ECM -- d_c = 15µm. The potential well-depth, ε, has been varied such that the cell's height matches that of the cell in the AFM indentation experiment (Hobson et al., 2020). The following table provides the values for the LJ parameters and the corresponding cell height in cell-spreading experiments:

σ(µm)	ε (zJ)	d_c(µm)	Cell Height (µm)
5	25000	15	13.9
5	50000	15	11.4
5	75000	15	11

In order to prepare the *'in silico'* cell for indentation simulations, we needed a more flattened cell and nucleus. We obtained the flattening with a nucleus-cytoplasm potential switched on with a well-depth half of that between cytoplasm-ECM. Once the desired cell height is achieved (~ 9 µm), the nucleus-cytoplasm potential was switched off and the cell was equilibrated again after spreading.

*Comment 2.8**. Equation 5 in the Methods give how the force for the indentation experiment is defined. Is the force between the indentation particle and every particle in the simulation? Or is the force only between the indentation particle and the immediate particles it is in contact with and 0 otherwise? If it is the former, that is not very physical and needs to be justified. The latter choise is the better implementation and the force should be given in the main text.*

Response 2.8
Thanks for opportunity to rectify and clarify this. The force is between the rigid indenter and the particles which come into contact with it. It is a repulsive force which only acts on the particles which come into contact with it. All interactions in SEM are local, which is why the modeling approach can lead to shape changes as emergent behaviour. In this case, the physics of an indentation experiment is respected because as soon as the indenter pushes its neighboring cell particles down, these particles push on the layer beneath, etc. This shifts the particle-particle distance away from their equilibrium distance leading to the development of stress in the cell/nucleus. We have provided this information in the updated Methods section.

Modification 2.8
In the Methods section, the following text has been modified:
"The force exerted on the indenter by each particle is determined by the following relationship:

$$F(r) = -K(r-R)^2 \qquad (5)$$

where K is the force constant, r is the distance between the particle and the center of the indenter, and R is the radius of the indenter."

to

"The force exerted on the indenter by particles in contact with the surface is determined by the following relationship:

$$F(r) = -K(r-R)^2 \qquad (6)$$

where K is the force constant, r is the distance between the particle and the center of the indenter, and R is the radius of the indenter. This force is only repulsive, i.e. F(r) =0 for r > R."

*Comment 2.9**. In the Cell migration through constriction section, why is the migration simulated via the addition and removal of particles? The particles are lagrangian particles, so isn't it more sensible to simulate migration by a force exerted between cytoplasmic and ECM particles, or an extra velocity added to the cytoplasmic particles (which is not a great modeling choice, but still better than deleting and adding particles). How is the adding/deleting of particles justified?*

Response 2.9
BIOPOINT inherits the convention introduced in earlier SEM work, including SEM++ by the lab of Petros Koumoutsakos (Milde, 2014). In this approach, we model migration by the addition and removal of particles to reflect the polymerization/depolymerization of the actin filaments which take place during cell migration. We have continued this framework for extracting subcellular mechanics, nuclear mechanics and cell-ECM interactions. In other words, we deal with the particle addition/removal during migration to preserve a mechanism akin to the cytoskeletal dynamics that drive migration. We expect it to become valuable in future studies when additional heterogenous particles can be added to simulate the actin cytoskeleton.

Modification 2.9
We have added the following text in Methods, in the subsection titled "Cell migration through constriction", to better describe this technique:
"We leveraged the simulation software SEM++ (Milde, 2014, [18]), where the particle ensembles move as we add a particle on one side of the cell (leading edge) and remove a particle from the other (trailing edge). This process reflects the polymerization/depolymerization of the actin filaments which take place during cell migration at the leading/trailing edge. The force balance described in Eq. 1 generates rapid re-adjustments of particle positions and velocities in the ensemble which restore cell rheology."

*Comment 2.10**. How are the sizes and total number of the particles chosen? Should it be according to the volume of the cell you are modeling? For example, if we were to use 1200 instead of 1000 particles to start, how would the parameters change? In other words, does using 1200 particles instead of 1000 require recalibration or can the parameters be mapped to new values?*

Response 2.10
We have varied the number of particles (Np) to 1200 to find out the answer to the reviewer's question and prepared a cell with Np = 10000 (ten times the original value) in order to further investigate this interesting question. The size of the nucleus remains 10% of cell size. We found that though the elasticity matches well, manifested by peaks in the force-time curves, the force relaxation associated with viscosity is not so well captured for Np = 10000. Thus, for a quantitative comparison of nonlinear deformation, a recalibration of parameter values is necessary if the number of particles changes drastically.
Our rationale for using Np = 1000 is as follows: It was recommended by the seminal SEM paper (Sandersius et al. 2008) that a minimum of 100 particles would be necessary to model the emergent cell shapes and properties. As we want to capture the emergent nuclear shapes and properties, we have chosen 100 nuclear particles and assumed that the nucleus comprises 10% of the cell, i.e. the cell consists of 1000 particles. This is the best choice in terms of computational time. The computational overhead increases with increase in the number of particles as the number of particle interactions increase and the timestep required is smaller. The increase in computational time makes it difficult to run the optimization procedure, which involves running many simulations serially.

Modification 2.10
We added the following text in the subsection titled "Calibration of Model Parameters via Single-Cell Indentation Experiments":
"One should carefully choose the resolution of BIOPOINT, and thus the number of particles in a cell, N_p. If N_p is varied, we might require sample preparation, nuclear phase separation and recalibration for a quantitative comparison with experiments. However, a small variation in N_p doesn't alter the indentation force-time curve significantly (SI, Fig. S7)."

In Discussion, we have also added a callout in this sentence:
"The number of particles used to model the nucleus could also be varied (thus varying particle size) to investigate its effect on emergent nuclear shape, although preparing a phase separated nucleus could be challenging with increase in the number of particles (See SI, Fig. S6, S7)."

The following text and figure are added in SI:
"**Varying the number of particles in a cell, N_p**

In order to investigate the effect of the number of particles, we have varied the N_p slightly to 1200 and also drastically to 10000. The size of the nucleus remains 10% of the cell in both these scenarios. Ideally, this requires preparing a new cell with revised number of particles, phase separating the nucleus and recalibrating the parameters. This process can become computationally expensive with a drastic increase in the number of particles. We have prepared two new cells with N_p = 1200, 10000 and derived the geometric parameter values, such as d_{eq}, particle viscosity, stiffness from the scaling relations of Eq. 3. For the bulk properties of cell and nucleus, we have continued with the values that we derived from optimization with N_p = 1000. We performed single cell indentation on each of these cells and plotted the force-time curves (Fig. S7). We found that the elasticity is well reproduced for both N_p =1200 and 10000, with a good agreement in the peak of the force-displacement curve at the end of the loading period of indentation. However, the force relaxation in the hold period for N_p = 1200 matches well with N_p = 1000, but the same is not true for N_p = 10000. Thus, for small variations in the number of particles, we could still continue with the same parameter values but for a drastic change, a recalibration is advisable to quantitatively capture nonlinear deformation. We recommend that the particle size should be chosen carefully before using our model, depending on the length scale that one wishes to probe in their applications."

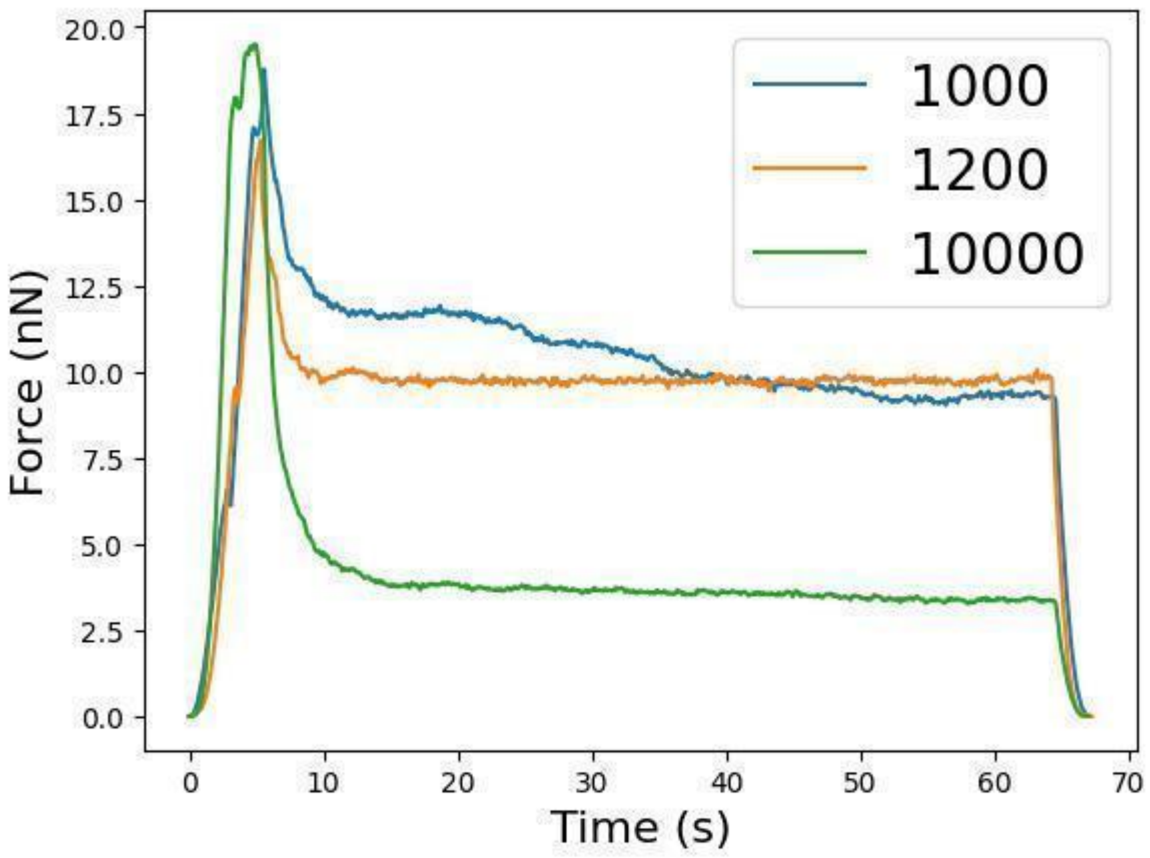

Fig. S7: Force versus time for single cell indentation simulations with different values of Np.

Comment 2.11. You mention that around 8% of cytoplasmic particles end up in the nucleus, that doesn't seem to be negligeable, even though it is not very large, can you comment on whether this is worrying and whether the 8% can be reduced.

Response 2.11
We start from a completely mixed ensemble of particles and allow the potentials to thermodynamically separate the phases over time. The mechanical properties of the nucleus are not very different from that of the cytoplasm. Therefore, the functional form of the potentials is the same with different parameter values. Under the circumstances, achieving a complete phase-separation is difficult with MD simulations (Zhang et al., J. Membr. Sci., 2022, vol. 661, 120922). Therefore, we considered the small number of isolated particles a good trade-off for computational load. We clarified this aspect in the manuscript.

Modification 2.11
In the subsection, titled "BIOPOINT uses heterogeneous discrete particle types to model the cell nucleus and ECM", after the following sentence:

"Still, after phase separation, fewer than 8% of cytoplasmic particles remained inside the nuclear boundary (Fig. 1C)."

we have added the following text

"Considering that we have started from a completely mixed configuration of equal-sized particles, the potentials of the two types of particles have the same functional form and there is no nuclear membrane, it is exponentially more difficult from a computational standpoint to obtain a better phase separation [34]."

*Comment 2.12**. Are the experiments you fit in the indentation experiment the same cell type as the ones you use later to fit the cell spreading experiments?*

Response 2.12

We chose to use a varied set of experiments to cross-validate the ability of BIO-POINT to capture emergent properties and make both quantitative and qualitative predictions. We found interesting dataset across different cell types: SKOV3 cells for indentation (Hobson et al. 2020), EC (Versaevel et al., 2012) and hMSC (Sarikhani et al., 2024) cells for spreading and MDA-MB-231 cells for migration through a constriction (Keys et al., 2024).

In our case, fitting was performed only on the indentation experiments: cell spreading and migration simulations were performed with these calibrated properties. What we were excited to show is that the properties obtained from a particular experiment and cell type can still semi-quantitatively or qualitatively capture the emergent behavior (eg, the cellular and nuclear deformation) of different cell types in different experiments. Of course, accurate predictions would need a re-calibration of the model (As discussed in 2.1) . We clarified our fitting and cross-validation strategy better in the Validation sub-section as described below.

Modification 2.12
In Discussion, after the sentence:

"BIOPOINT's validation across multiple experimental conditions supports its application for investigating nuclear mechanics in morphogenesis."

we have added the following text

"We calibrated parameters on a particular cell type and cross-validated it across different cell types depending on the data we found in the literature. While the semi-quantitative agreements (Fig. 3) indicate robustness of our emergent property predictions, high-accuracy simulations may need the model to be calibrated and validated against data collected on the same cell type, especially as mechanical properties may vary across cell types, but also in the same cell type in different situations [40–42]. A discussion on how the parameter values are expected to change in different cell types is provided in SI."

Comment 2.13. Related to 12 and 10, could you mention what range of values for the parameters could be expected if the cell types were to change?

Response 2.13

Across adherent mammalian cells, AFM and microrheology studies show that the apparent cell and nuclear elastic moduli and viscosities that BIOPOINT's spring (K) and dashpot (η) parameters coarse-grain vary by about one to two orders of magnitude between cell types. Apparent cell Young's moduli typically range from ~0.1–2 kPa for soft epithelial and cancer cells up to ~10–20 kPa for stiffer endothelial or fibroblast cells, while nuclear moduli span ~0.2–10 kPa and are usually 2–4× higher than the surrounding cytoplasm (Garcia et al, Nanoscale, 2020). Cytoplasmic viscosities inferred from micropipette aspiration, rotational microrheology and particle tracking lie broadly between ~1 and 10^3 Pa·s, with nuclear or perinuclear viscosities again typically a few-fold higher (Dessard et al, Nanoscale Adv, 2024). Therefore, if the cell type were changed, we would expect BIOPOINT's calibrated cytoplasmic and nuclear stiffnesses (κ_0, κ_{nuc}) and viscosities (η_0, η_{nuc}) to shift by at most ~0.1–10× relative to the SKOV3 values reported in Table 1, while preserving a nuclear-to-cytoplasmic stiffness ratio of order 2–4 and viscosity ratio of order 2–5; these ranges are consistent with the spread of experimental rheological measurements across epithelial, fibroblast, endothelial and cancer cell lines.

Modifications 2.13:
We have added the following sentence in Discussions:
"A discussion on how the parameter values are expected to change in different cell types is provided in SI."

Furthermore, we have included a discussion regarding different cell types in SI where we have also added the Table ST1:

Expected ranges of parameter values for different cell types

In BIOPOINT, the key rheological parameters are the cytoplasmic and nuclear spring constants (κ_0, κ_{nuc}) and viscosities (η_0, η_{nuc}) that define the pairwise interaction potentials between particles (Tables 1 and 2). These quantities are not direct continuum moduli, but were calibrated by matching AFM indentation force–time curves against SKOV3 cells using a Kelvin-Voigt-like viscoelastic response at the coarse-grained level. To estimate how these parameters would change for other cell types, we reviewed experimental measurements of apparent elastic moduli and viscosities for cytoplasm, cortex, and nuclei in representative mammalian cells.

Apparent cell stiffness. AFM-based indentation and nanorheology studies routinely find that adherent mammalian cells span roughly 1–2 orders of magnitude in apparent Young's modulus, depending on cell type, substrate, probe geometry, and loading rate. A high-throughput nanorheology analysis of NIH 3T3 fibroblasts reported apparent moduli $E_0 \approx 0.6$–1.3 kPa over a broad range of indentation velocities, with modest stiffening at higher speeds [3]. In contrast, AFM nanoindentation of vascular endothelial cells (bEnd.3) using a conical tip and viscoelastic Neo-Hookean modeling yielded effective elastic moduli in the 16–20 kPa range, again with some loading-rate dependence [4]. Reviews of AFM measurements across many cell lines support a typical range of ~0.1–2 kPa for many epithelial and cancer cells, with fibroblasts and endothelial cells often an order of magnitude stiffer when probed at the cortex [3]. These values define the continuum "target" behavior that BIOPOINT's κ_0 is designed to reproduce. Because
κ_0 scales approximately linearly with the apparent indentation modulus for a fixed geometry, we expect κ_0 for other adherent cell types to vary by ~0.1–10× around the SKOV3-fitted value.

Nuclear stiffness. Multiple AFM and micromanipulation studies concur that the nucleus is typically stiffer than the surrounding cytoplasm and that its modulus is strongly tuned by lamin A/C and chromatin state. A recent study on live cells reported cytoplasmic stiffness of ~0.5–3 kPa and nuclear stiffness in the ~1–10 kPa range, depending on lamin distribution and substrate conditions. Direct AFM probing of exposed endothelial nuclei found nuclear regions with mean modulus ~5.6 kPa compared to ~1.5 kPa in non-nuclear regions of the same cells (nucleus/cytoplasm stiffness ratio ≈3–4) [5]. At the

softer end, AFM measurements on MDA-MB-231 breast cancer cells showed nuclear stiffness values of order 10^2 Pa, which increased roughly twofold upon histone deacetylase inhibition, again preserving a nucleus-to-cytoplasm stiffness ratio of a few-fold [6]. These data imply that, across adherent mammalian cells, nuclear moduli occupy roughly 0.2–10 kPa, with a fairly robust nucleus/cytoplasm stiffness ratio of ~2–4. BIOPOINT's optimized parameters ($\kappa_{nuc} \approx 3.8\times\kappa_0$) fall squarely in this range, and we therefore expect κ_{nuc} to vary on the same order as κ_0 (roughly 0.2–10× its SKOV3 value) when targeting different cell types, while maintaining a stiffness ratio in the 2–4 band. This justifies the prior range 1–3 used for the coupling parameter k_{12} in our uncertainty quantification.

Cytoplasmic viscosity and relaxation times. Cytoplasmic viscosity has been quantified using a variety of techniques, including micropipette aspiration, particle-tracking microrheology, and magnetic rotational spectroscopy (MRS), and exhibits large variability across cell types and intracellular locations. MRS measurements in mammary epithelial cells (MCF-10A) and breast cancer lines (MCF-7, MDA-MB-231) reported cytoplasmic viscosities in the range 10–70 Pa·s with viscoelastic moduli of 30–80 Pa, yielding characteristic relaxation times $\tau = \eta/G$ on the order of 0.1–3 s [7]. A micropipette-based analysis of hundreds of individual lung tumor cells (H1299, A549) treated as Newtonian drops found much broader single-cell viscosity distributions: for H1299 cells, quartiles spanned ~17, 42, and 110 Pa·s, while A549 cells exhibited medians near 490 Pa·s with upper quartiles above 10^3 Pa·s [8]. Earlier work on white blood cells and embryonic systems reported cytoplasmic viscosities ranging from a few Pa·s up to several hundred Pa·s, depending on probe size and timescale [9]. Together, these data suggest an "operational" cytoplasmic viscosity range of ~1–10^3 Pa·s for many mammalian cells, with differences of 5–10× between related lines (e.g., low- vs high-metastatic cells) being common [10]. Nuclear viscosities are less well constrained experimentally, but chromatin-focused rheology and HP1α perturbation experiments consistently show that chromatin-rich nuclear regions are more dissipative than the surrounding cytoplasm, leading to relaxation times that are typically a few-fold longer [11]. In BIOPOINT, η_0 and η_{nuc} are tuned to match the AFM force–relaxation curve of SKOV3; the experimental ranges above imply that re-fitting η_0 and η_{nuc} for different cell types should mainly involve ~0.1–10× rescaling.

Link to BIOPOINT parameter ranges. The discrete spring and dashpot parameters in BIOPOINT ($\kappa_0, \eta_0, \kappa_{nuc}, \eta_{nuc}$) are not directly equal to continuum Young's moduli and viscosities, but for a fixed particle discretization and indentation geometry they scale approximately linearly with the corresponding apparent moduli. Our SKOV3 calibration targets AFM data that fall within the low-kPa and 10–100 Pa·s regimes discussed above [12]. Taken together, the literature therefore supports using prior ranges of roughly 0.1–10× the SKOV3-fitted values for both K and η when adapting BIOPOINT to other adherent mammalian cell types. More extreme phenotypes (very soft embryonic cells, highly stiffened myofibroblasts, etc.) may require excursions up to ~2 orders of magnitude, but even in those cases the nucleus/cytoplasm stiffness ratio and the qualitative ordering $\eta_{nuc} > \eta_0$ are expected to hold. The ranges summarized in Table ST1 can thus serve as biologically grounded priors or bounds when re-calibrating BIOPOINT for new cell lines or conditions.

Table ST1. BIOPOINT rheological parameters and expected variation across cell types, with representative experimental ranges.

BIOPOINT parameter	Physical interpretation	SKOV3-fitted value (this work)	Expected variation for other adherent mammalian cells*	Representative experimental ranges / examples (continuum quantities)	Key refs
κ_0 [N·m⁻¹]	Effective spring constant for cytoplasmic + cortical network, controlling cell indentation response	1.2×10⁻² N·m⁻¹	~0.1–10× SKOV3 (≈10⁻³–10⁻¹ N·m⁻¹)	Apparent cell Young's modulus E_cell ≈ 0.1–2 kPa for many epithelial/cancer cells; NIH 3T3 fibroblasts ~0.6–1.3 kPa; vascular endothelial cells up to ~16–20 kPa depending on loading rate.	[3,4]
η_0 [N·s·m⁻¹]	Effective viscous damping between cytoplasmic particles (Kelvin-Voigt–like dashpot)	5.0×10⁻² N·s·m⁻¹	~0.1–10× SKOV3 (≈5×10⁻³–5×10⁻¹ N·s·m⁻¹)	Cytoplasmic viscosities from microrheology/micropipette typically ~10–70 Pa·s for mammary epithelial/cancer cells (MCF-10A/MCF-7/MDA-MB-231), with single-cell distributions spanning ~0.6–500 Pa·s; lung cancer cells (H1299, A549) show medians from ~40 to ~500 Pa·s and upper quartiles above 10³ Pa·s.	[7,8]
κ_{nuc} [N·m⁻¹]	Spring constant for nuclear phase, capturing nuclear stiffness	4.5×10⁻² N·m⁻¹	0.2–10× SKOV3 (≈10⁻²–5×10⁻¹ N·m⁻¹)	Nucleus generally stiffer than cytoplasm: cytoplasmic moduli ~0.5–3 kPa vs nuclear moduli ~1–10 kPa in many cell types;[13] MDA-MB-231 nuclei can be as soft as ~0.1–0.2 kPa under some perturbations, while exposed endothelial nuclei reach ~5–6 kPa.[6]	[5,6,13]

η_{nuc} [N·s·m⁻¹]	Viscous damping within the nuclear phase	2.1×10^{-1} N·s·m⁻¹	~0.2–10× SKOV3 (≈4×10^{-2}–2 N·s·m⁻¹)	Cytoplasmic MRS measurements give η ≈ 10–70 Pa·s with elastic moduli 30–80 Pa, corresponding to relaxation times τ ≈ 0.1–3 s; nuclear or chromatin-dominated regions are typically a few-fold more viscous.[7]	[7,10,11]
k_{12} [–]	Relative strength of nucleus–cytoplasm interaction potential	1.51	Likely 1–3	AFM and FEA studies consistently report nucleus/cytoplasm stiffness ratios between ~2 and 4 in many adherent cell types.[13]	[13]
λ [–]	Dimensionless tuning coefficient	0.75	Expected near 0.5–1 for most cell types; primarily numerical/phenomenological rather than directly tied to a single rheological observable	No direct experimental analog.	BIOPOINT (this work)

*Order-of-magnitude estimates based on published ranges of apparent moduli and viscosities; precise values should be re-identified by re-fitting BIOPOINT to AFM/microrheology data for the specific cell type of interest.

Comment 2.14. You say "the projected area expanding proportionally to the adhesion potential", I don't think you show proportionality, you show that one increases as the other increases, which is not necessarily a proportional relationship.

Response 2.14
We have now modified the above sentence.

Modification 2.14
The text has been changed the sentence:
"Increasing the adhesion strength resulted in greater cell spreading (Fig. 1D, right), with the projected area expanding proportionally to the adhesion potential (Fig. 1E, Fig. S1)."

to

"Increasing the adhesion strength resulted in greater cell spreading (Fig. 1D, right), with the projected area being more for a higher value of ε, which corresponds to well depth of the LJ potential (Fig. 1E, Fig. S1)."

*Comment 2.15**. Are the immobilized peripheral cytoplasmic particles used in the indentation experiment necessary? Can't the interaction between cytoplasmic particles and the ECM and/or the attraction between cytoplasmic particles themselves keep the cell from spreading too much? Please justify the immobile particles.*

Response 2.15
The interaction between cytoplasmic-ECM particles and the cytoplasmic-cytoplasmic particles is designed to balance the amount of cell spreading on a large ECM substrate (where the cell spreading is not constrained by the geometry of the ECM). In order to constrain the cell from sliding/spreading further during the indentation experiment, the peripheral particles are immobilized. This immobilization is assumed to capture the effect of the focal adhesions which prevent a cell from spreading during an indentation experiment. Since this sliding/spreading happens specifically during indentation experiments, we do not use this observation to calibrate parameter values pertaining to the cell's general properties and cell-ECM interactions.

Modification 2.15
The following sentence is added in Methods in the subsection titled "Indentation":
"The peripheral particles were immobilized so that the cell doesn't spread further when it is being indented, which is achieved experimentally due to the focal adhesions of the cell on the ECM substrate. The effect of peripheral constraint on the force-time curve can be seen in Fig. S8."

We have added Supplementary Fig. S8 to demonstrate what happens if the peripheral particles are not immobilized.

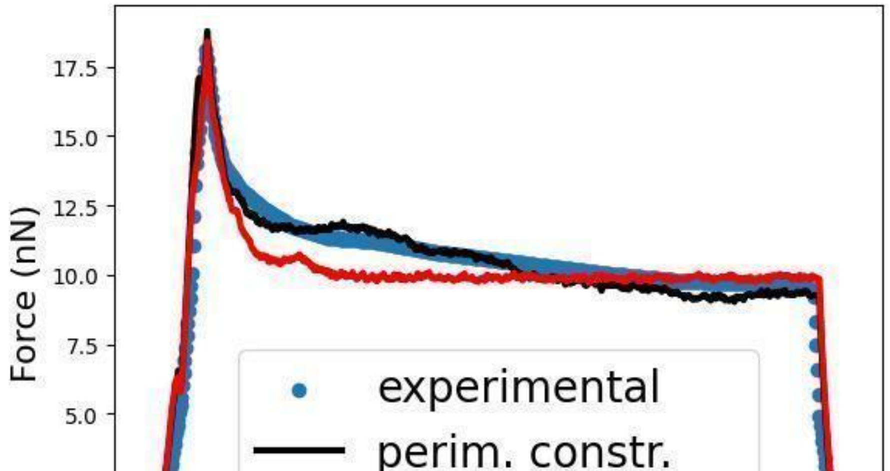

Comment 2.16. As a matter of style, the plot in Figure 4F should just be a line plot without the filling. It currently looks unnecessarily complicated.

Response 2.16
We have modified Fig. 4F as per the reviewer's suggestion. The filling has been removed.

Modification 2.16
Figure 4F has been modified.

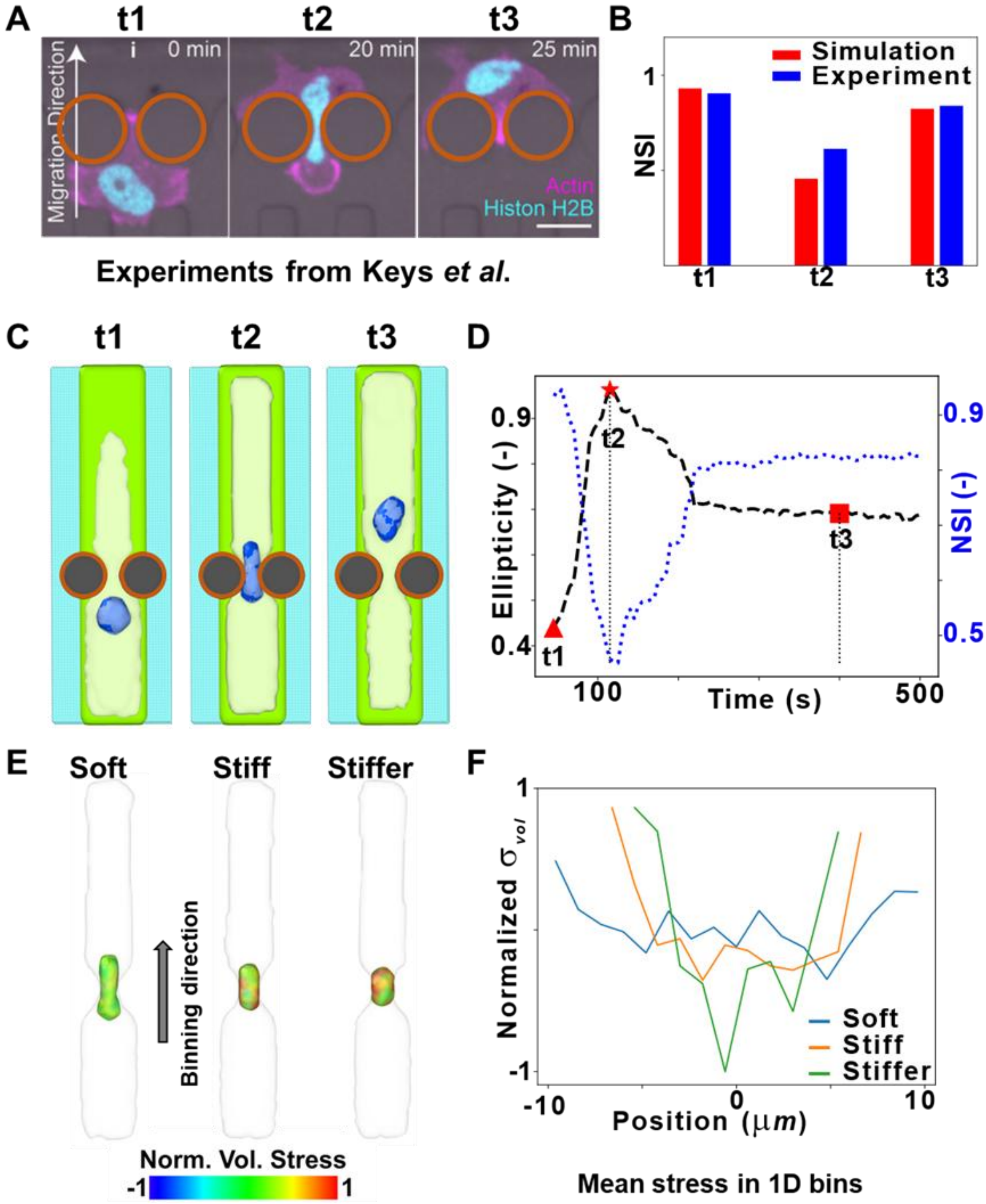

Comment 2.17. Could the discrepancy between the experiment and your nuclear shape (Figure 3G) be due to using a nucleus in your model that's too small? It looks like the nucleus doesn't need to deform much to fit into the ECM pattern, if the nucleus was larger, it might deform more.

Response 2.17

The size of the nucleus is an important factor in determining whether the nucleus will deform or not. However, in this case, the nucleus is not too small to deform. This can be clearly seen the Fig. S3-B (below). With the addition of the (2-3) potential between nuclear-ECM particles, the nucleus changes shape drastically (NSI ~ 0.3). The nucleus in this cell is of the same size (100 particles, 10% of cell) as the ones with which Fig. 3G has been produced. Thus, the potentials have an important role in determining the nuclear deformation in such simulations. However, in order to test the reviewer's suggestion, we designed simulations of cells with larger nucleus. These cells comprise 200/800 particles for nucleus/cytoplasm, i.e. nuclear size is 20% of cell size. We find that the nucleus doesn't deform more than the cell with smaller nucleus (100/900 particles) as can be appreciated in the figure below.

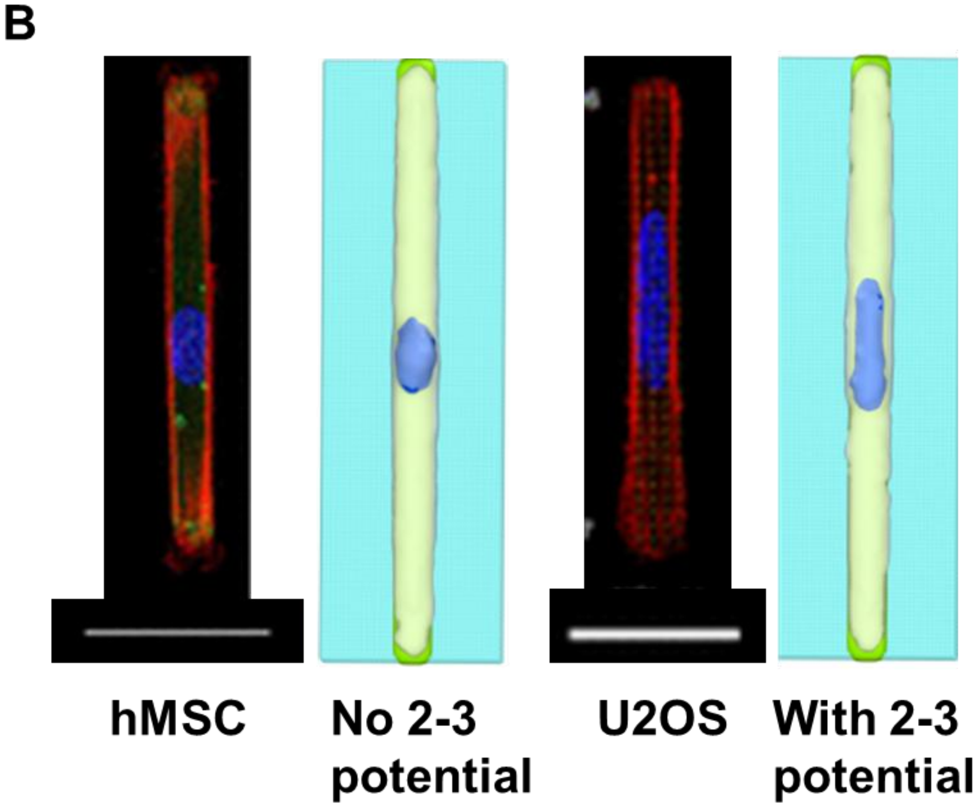

Modification 2.17

We have added the following sentence in Methods, in the subsection titled "Cell spreading simulations on ECM patterns":

"We chose 100/900 nuclear/cytoplasmic particles in order to have the minimum number of nuclear particles to reproduce emergent nuclear shapes [16], but conducted additional simulations with 200 nuclear particles representing the nucleus (see SI, Fig. S6)."

We have added the following text in the SI:

"In order to investigate the effect of nuclear size, we have designed a cell with twice the nuclear size, i.e. 200 nuclear particles and 800 cytoplasmic particles. We have performed cell spreading on the various ECM patterns that were used in Fig. 3. We repeated the analyses of cell shape and nuclear shape for this cell with larger nucleus. We didn't observe that the nucleus deforms much more than the smaller nucleus comprising 100 particles. This suggests that in the simulation scenarios studied here the

potentials governing the nuclear, cytoplasmic and ECM particles and the interplay between them plays a bigger role in nuclear deformation than nuclear size."

We have added supplementary figure S6 to show the effect of a bigger nucleus with 200 particles.

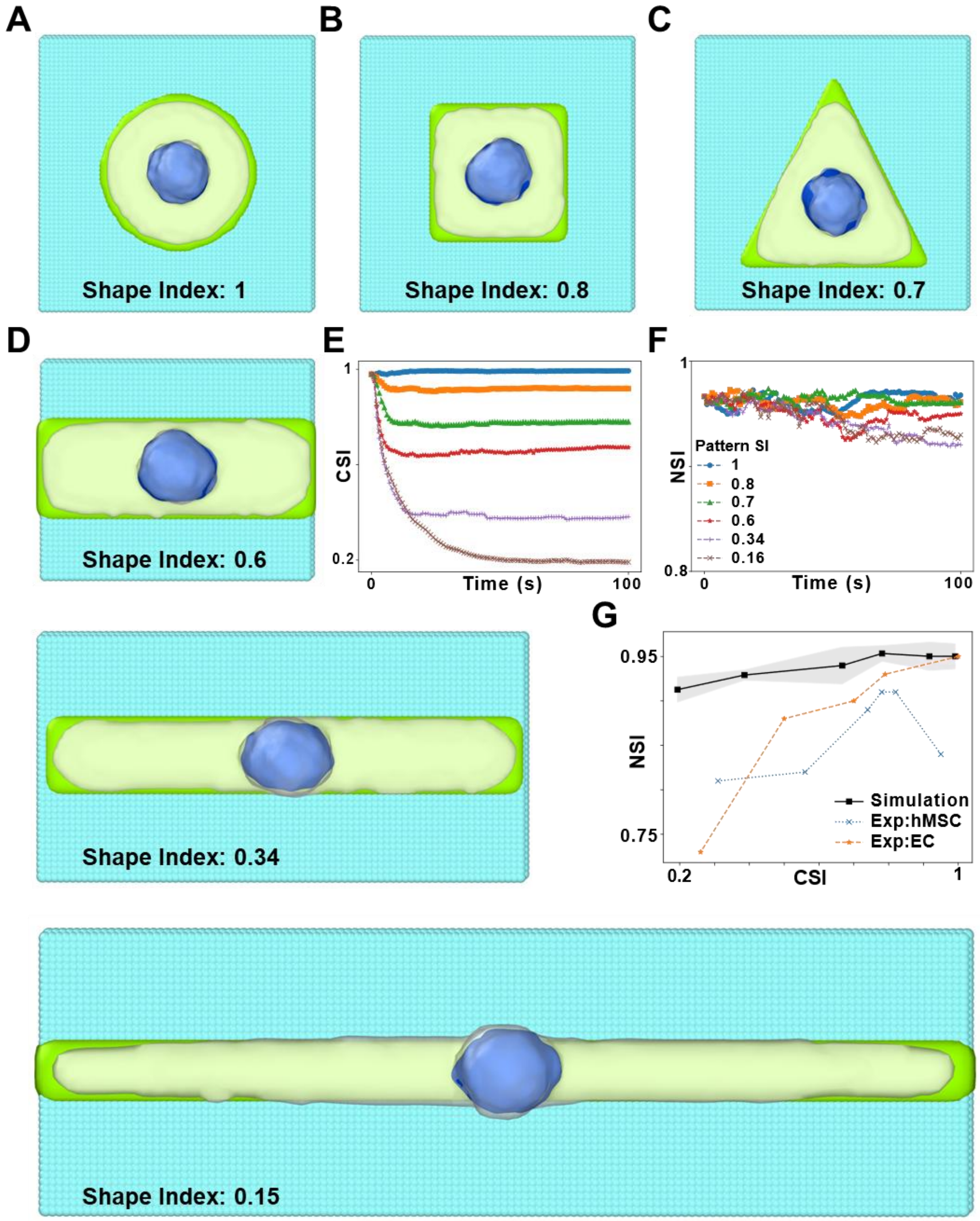

Supplement: Attachment — Submitted filename: BIOPOINT_Response to reviewers.pdf [file pcbi.1014113.s008.pdf]
