# Supplementary material for: BIOPOINT: A particle-based model for probing nuclear mechanics and cell-ECM interactions via experimentally derived parameters
Source: PLoS Comput Biol. 2026 Mar 31;22(3):e1014113. doi: 10.1371/journal.pcbi.1014113 (PMC13056257; doi:10.1371/journal.pcbi.1014113)

We thank the editors and the reviewers for giving us another opportunity to improve our manuscript. We present below a detailed response to the received feedback, as well as a summary of the modifications implemented in the manuscript.

Additional Editor Comments:

Comment E1

While the reviewers are generally positive about the improvements, Reviewer 2 has identified critical instances where the interpretation of the results remains overclaimed, specifically regarding the validation against different cell types and the sensitivity of particle numbers.

Response E1

We understand Reviewer 2's concerns and have addressed them in the revised manuscript as explained below. Additionally, we have changed Figure 2A in response to copyright considerations. We added a schematic instead of using the original image.

Modification E1

Old Fig. 2A

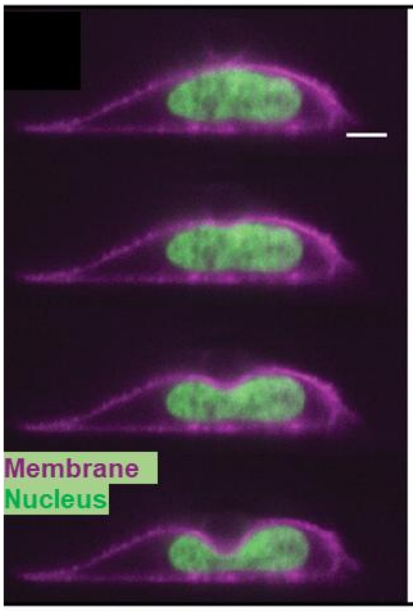

New Fig 2A

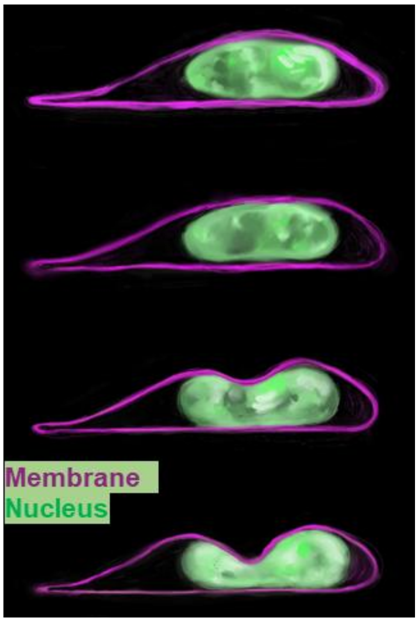

Reviewers' comments:

Reviewer's Responses to Questions
Reviewer #1

Comment 1.0

In the revised manuscript, the authors addressed most concerns well. I have a few remaining comments.

Response 1.0
We appreciate the reviewer's comments and address the remaining comments below.

Modification 1.0
No modifications.

Comment 1.1

1) In the revised introduction, there is a long section starting with "Prior SEM implementations have typically represented the nucleus..." and ending in "...influences cell spreading and mechanosensing". This section should include references to these prior SEM implementations.

Response 1.1
We have added references to these prior SEM implementations.

Modification 1.1
Old text:
"Prior SEM implementations have typically represented the nucleus as a single rigid particle, which fails to account for nuclear deformability and the role of intracellular forces in nuclear positioning and chromatin remodeling. Similarly, the ECM has generally been modeled as an implicit solvent rather than a discrete, separate environment directly interacting with cells. Finally, SEM parameters were traditionally derived by homogenizing bulk elastic stiffness and viscosity of non-adherent cells, leading to model parameters that might not be relevant for cell-ECM interactions during morphogenesis. As a result, current SEM frameworks cannot accurately model processes such as nuclear deformation during confined migration or how ECM geometry influences cell spreading and mechanosensing."

New text:
"Prior SEM implementations have typically represented the nucleus as a single rigid particle **[18,19]**, which fails to account for nuclear deformability and the role of intracellular forces in nuclear positioning and chromatin remodeling. Similarly, the ECM has generally been modeled as an implicit solvent rather than a discrete, separate environment directly interacting with cells **[18,19]**. Finally, SEM parameters were traditionally derived by homogenizing bulk elastic stiffness and viscosity of non-adherent cells **[16,18,19]**, leading to model parameters that might not be relevant for cell-ECM interactions during morphogenesis. As a result, current SEM frameworks cannot accurately model processes such as nuclear deformation during confined migration or how ECM geometry influences cell spreading and mechanosensing."

References:
16. Sandersius SA, Newman TJ. Modeling cell rheology with the Subcellular Element Model. Phys Biol. 2008;5: 015002. doi:10.1088/1478-3975/5/1/015002
17. Roeder AHK, Tarr PT, Tobin C, Zhang X, Chickarmane V, Cunha A, et al. Computational morphodynamics of plants: integrating development over space and time. Nat Rev Mol Cell Biol. 2011;12: 265–273. doi:10.1038/nrm3079
18. Milde F, Tauriello G, Haberkern H, Koumoutsakos P. SEM++: A particle model of cellular growth, signaling and migration. Comp Part Mech. 2014;1: 211–227. doi:10.1007/s40571-014-0017-4

19. Chattaraj S, Torre M, Kalcher C, Stukowski A, Morganti S, Reali A, et al. SEM2: Introducing mechanics in cell and tissue modeling using coarse-grained homogeneous particle dynamics. APL Bioengineering. 2023;7: 046118. doi:10.1063/5.0166829

Comment 1.2

2) The authors write "cell mechanics is lost (e.g., Potts models [22])", implying that the cellular Potts model does not represent cell mechanics. First, the Potts model is not the same as the cellular Potts model, so please adjust the wording. Second, the statement is factually incorrect. Cellular Potts models do represent cell mechanics at the cell membrane as the balance of forces between cell pressure, surface tension due to actin cortex contractility, and interfacial adhesion (Magno et al. 2015). Energy is related to force via integration, and interconversions within the cellular Potts formalism were shown by Rens & Edelstein-Keshet (2019). Similar energy-based formulations are also used in particle-based models (including molecular dynamics-based methods) and vertex models (Fletcher et al. 2014), which the authors agree can represent aspects of cell mechanics. I suggest rewording to clarify that internal or cytosolic cell mechanics are not represented, which is true for both cellular Potts models and vertex based models.

References:
Magno, R., Grieneisen, V.A. & Marée, A.F. The biophysical nature of cells: potential cell behaviours revealed by analytical and computational studies of cell surface mechanics. BMC Biophys 8, 8 (2015). https://doi.org/10.1186/s13628-015-0022-x
Rens EG, Edelstein-Keshet L (2019) From energy to cellular forces in the Cellular Potts Model: An algorithmic approach. PLOS Computational Biology 15(12): e1007459. https://doi.org/10.1371/journal.pcbi.1007459
Fletcher, Alexander G., et al. "Vertex models of epithelial morphogenesis." Biophysical journal 106.11 (2014): 2291-2304. https://doi.org/10.1016/j.bpj.2013.11.4498

Response 1.2
We apologize for the confusion. We meant to say that a spatially varying description of cell and tissue mechanics is lost in CPM and the vertex model, as the reviewer correctly summarized. We edited the text accordingly and also added new references, highlighted in yellow below.

Modification 1.2
Old text:
"Discrete models naturally capture biological processes but are costly, and cell mechanics is lost (e.g., Potts models [22]) or limited to cell membranes (e.g., vertex modeling [7])."
New text:
"Discrete models naturally capture biological processes and are effective in resolving cell mechanics at the cell membrane as a balance of forces [22]. Energy is related to force via integration and interconversions within the Cellular Potts formalism [23] or vertex models [24]. However, the cell mechanics in these cases is limited to cell membranes [7,25]."

References:
7. Kim S, Pochitaloff M, Stooke-Vaughan GA, Campàs O. Embryonic tissues as active foams. Nat Phys. 2021;17: 859–866. doi:10.1038/s41567-021-01215-1
21. Maxian O, Mogilner A, Strychalski W. Computational estimates of mechanical constraints on cell migration through the extracellular matrix. Rao CV, editor. PLoS Comput Biol. 2020;16: e1008160. doi:10.1371/journal.pcbi.1008160
22. Magno R, Grieneisen VA, Marée AF. The biophysical nature of cells: potential cell behaviours revealed by analytical and computational studies of cell surface mechanics. BMC Biophys. 2015;8: 8. doi:10.1186/s13628-015-0022-x
23. Rens EG, Edelstein-Keshet L. From energy to cellular forces in the Cellular Potts Model: An algorithmic approach. Gallo J, editor. PLoS Comput Biol. 2019;15: e1007459. doi:10.1371/journal.pcbi.1007459
24. Fletcher AG, Osterfield M, Baker RE, Shvartsman SY. Vertex Models of Epithelial Morphogenesis. Biophysical Journal. 2014;106: 2291–2304. doi:10.1016/j.bpj.2013.11.4498

25. Wortel IMN, Niculescu I, Kolijn PM, Gov NS, De Boer RJ, Textor J. Local actin dynamics couple speed and persistence in a cellular Potts model of cell migration. Biophysical Journal. 2021;120: 2609–2622. doi:10.1016/j.bpj.2021.04.036

Comment 1.3

3) In the caption of Figure 1 F, the authors mention "The cell membrane is white". Please reword to clarify that there is no actual cell membrane; the renders show the peripheral particles as white (or something along these lines—avoid confusion about any membrane particles).

Response 1.3
The sentence in the caption of Fig. 1F has been reworded as indicated below.

Modification 1.3
Old text:
"The cell membrane is white, and the nucleus is color-coded according to the per-particle stress."
New text:
"For visualization purposes, surface meshes have been generated on top of the respective particles, representing the cell membrane (in white) and the nuclear membrane (color-coded according to the per-particle stress)."

Comment 1.4

4) How often do you observe that some of the nucleus particles are exposed to the medium without any peripheral cytosolic particles surrounding them? At least in the OVITO renders and supplementary movies, this seems to occur fairly often. Is this a source for concern? Could this explain why adding a 2-3 potential between nucleus and ECM leads to greater nuclear deformation? Should this be addressed in future studies (-> discussion material)?

Response 1.4
In highly flattened spreading geometries (2D substrate-guided spreading), BIOPOINT's current coarse-grained representation lacks an explicit cortical shell/perinuclear cytoskeletal layer that would enforce a uniformly thick cytoplasmic envelope around the nucleus. As a result, local thinning of the cytoplasm around the nucleus can occur, making nuclear particles appear partially exposed in particle-based renders. This also clarifies why adding an exploratory nucleus–ECM coupling (2–3 interaction) can increase nuclear deformation in those conditions. However, the flattened nucleus would still be exposed to the ECM substrate because the nuclear and cell membranes are absent. We now state this limitation explicitly and frame the 2–3 interaction as a sensitivity analysis, motivating future extensions (explicit cortex/cytoskeletal constraints).

Modification 1.4

- **(Methods; "Preparation of multi-particle nucleus")**
  Old text:
  "The biasing force on the nucleus towards cell center, along with the superior cytoplasmic-nuclear adhesion compared to the cytoplasmic-cytoplasmic adhesion (Fig. S5), ensures that it is always surrounded by cytoplasmic particles."
  New text:
  "The biasing force on the nucleus towards the cell center, along with the superior cytoplasmic–nuclear adhesion compared to cytoplasmic–cytoplasmic adhesion (Fig. S5), favors the nucleus remaining embedded within the cytoplasmic particle ensemble. In highly flattened 2D spreading simulations, local thinning of the cytoplasmic layer around the nucleus can occur; we discuss this limitation and its implications for nucleus–ECM coupling in the Discussion."
- **(Results; cell spreading section, paragraph discussing mismatch of extreme nuclear elongation; final sentence referencing Fig. S3)**
  Old text:
  "However, these simulations failed to capture the drastic nuclear elongation in some experimental conditions, which might require additional potentials to match the stress state in these cell lines (See Fig. S3)."
  New text:
  "However, these simulations failed to capture the most extreme nuclear elongation reported in some experimental conditions, which likely reflects missing force-transmission mechanisms (e.g., actin-mediated perinuclear compression). We therefore tested a weak nucleus–ECM interaction implemented as an attractive–repulsive Lennard-Jones potential (2–3 potential; Fig. S3), which can increase nuclear deformation in highly elongated patterns."
- **(Discussion; paragraph starting "Our cell spreading simulations confirmed…")**
  Old text:
  "Our cell spreading simulations confirmed that ECM geometry influences nuclear shape (Fig. 3), though BIOPOINT underestimated nuclear elongation in high-aspect- ratio ECM conditions. This discrepancy likely arises from the absence of actin filament- mediated nuclear compression, which is known to contribute to nuclear deformation [29]. Incorporating polymeric chains to model actin filaments could improve BIOPOINT's ability to capture cytoskeletal contributions to nuclear reshaping [44,45]."
  New text:

"Our cell spreading simulations confirmed that ECM geometry influences nuclear shape (Fig. 3), though BIOPOINT underestimated nuclear elongation in high-aspect-ratio ECM conditions. This discrepancy likely arises from the absence of actin filament–mediated nuclear compression, which is known to contribute to nuclear deformation [32]. In addition, because BIOPOINT currently lacks an explicit actin cortex/perinuclear shell, highly flattened spreading geometries can lead to local thinning of the cytoplasmic layer around the nucleus, which can make the nucleus appear partially exposed in particle-based renders. This limitation also increases sensitivity to any added nucleus–ECM coupling (e.g., the exploratory 2–3 interaction in Fig. S3). Incorporating polymeric chains to model actin filaments and/or an explicit cortical/perinuclear constraint could improve BIOPOINT's ability to capture cytoskeletal contributions to nuclear reshaping [47,48]."

References:

32. Versaevel M, Grevesse T, Gabriele S. Spatial coordination between cell and nuclear shape within micropatterned endothelial cells. Nat Commun. 2012;3. doi:10.1038/ncomms1668

47. Lykov K, Nematbakhsh Y, Shang M, Lim CT, Pivkin IV. Probing eukaryotic cell mechanics via mesoscopic simulations. Nie Q, editor. PLoS Comput Biol. 2017;13: e1005726. doi:10.1371/journal.pcbi.1005726

48. Lüchtefeld I, Pivkin IV, Gardini L, Zare-Eelanjegh E, Gäbelein C, Ihle SJ, et al. Dissecting cell membrane tension dynamics and its effect on Piezo1-mediated cellular mechanosensitivity using force-controlled nanopipettes. Nat Methods. 2024;21: 1063–1073. doi:10.1038/s41592-024-02277-8

Comment 1.5

5) Is the 2-3 potential you test in Figure S3 purely repulsive? Or what form does it have? Perhaps it would be good to show it in a sub-panel in the figure.

Response 1.5
The 2-3 potential is shown in Figure S5, and it is an attractive-repulsive Morse potential weaker than the 1-3 potential. We have added a subpanel to plot this potential. The modifications in SI are highlighted in yellow.

Modification 1.5
Old text:
"However, these simulations failed to capture the drastic nuclear elongation in some experimental conditions, which might require additional potentials to match the stress state in these cell lines (See Fig. S3)."
New text:
"However, these simulations failed to capture the most extreme nuclear elongation reported in some experimental conditions, which likely reflects missing force-transmission mechanisms (e.g., actin-mediated perinuclear compression). We therefore tested a weak nucleus–ECM interaction implemented as an attractive–repulsive Lennard-Jones (12-6) potential (2–3 potential; Fig. S3), which can increase nuclear deformation in highly elongated patterns."

Old S3:

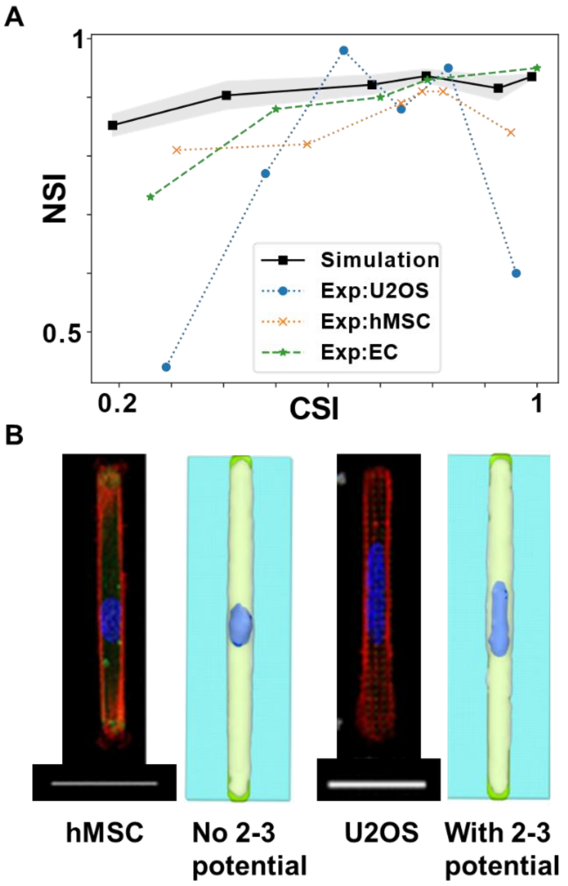

Figure S3. Effect of the nuclear-ECM potential. A) Plot of NSI versus CSI for representative simulations and experimental results from the literature for cell types: EC [2], hMSC and U2OS [1]. B) Comparison of cell spreading simulations versus experimental results for hMSC and U2OS cells [23]. Experimental representative widefield images from Sarikhani et al. [1] have ECM in green, actin in red

and nucleus in blue. Scale bar is 50 µm and 40 µm for hMSC and U2OS cells respectively. The simulations of cell spreading are without and with nucleus-ECM (2-3) potential, respectively.

New S3:

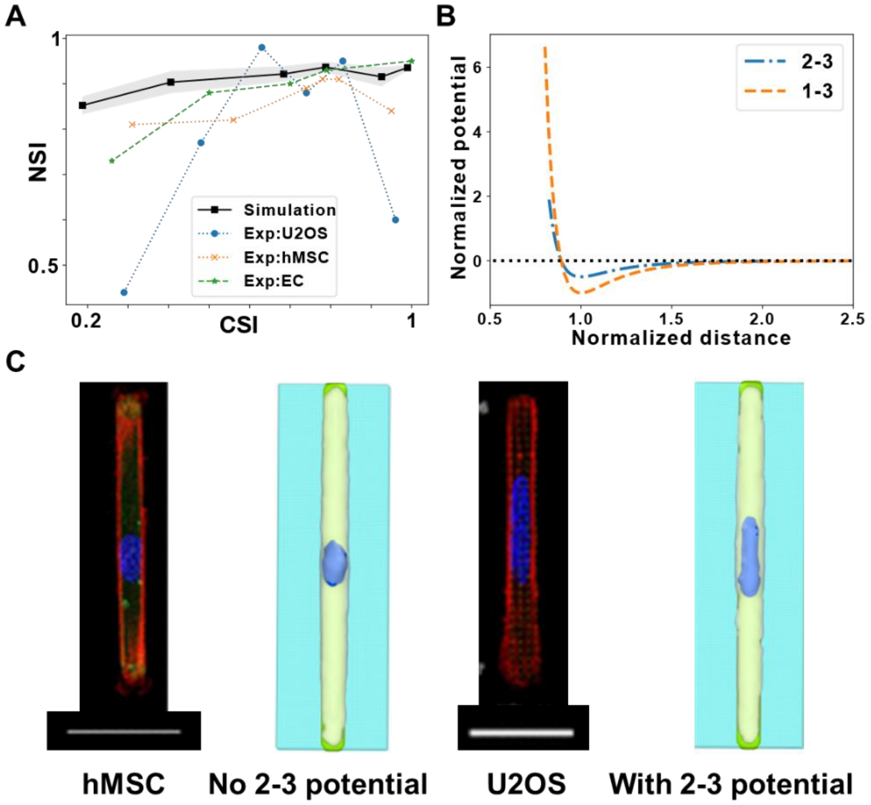

Figure S3. Effect of the nuclear-ECM potential. A) Plot of NSI versus CSI for representative simulations and experimental results from the literature for cell types: hMSC, U2OS [2] and EC [3]. B) Plot of normalized potential (LJ 12-6) versus normalized distance for cytoplasm-ECM (1-3) and nucleus-ECM (2-3) interactions. C) Comparison of cell spreading simulations versus experimental results for hMSC and U2OS cells [2]. Experimental representative widefield images from Sarikhani et al. [2] have ECM in green, actin in red and nucleus in blue. Scale bar is 50 µm and 40 µm for hMSC and U2OS cells respectively. The simulations of cell spreading are without and with nucleus-ECM (2-3) potential, respectively.

Reviewer #2

Comment 2.0

*The manuscript is now written with much higher clarity in terms of the Methods. Most of my questions have been addressed. However, I *strongly suggest* the following 2 points be addressed in the Results sections before acceptance for publication.*

This is to make the results clearer, more accurate, and more useful for the Plos Comp Bio audience.

Response 2.0
We appreciate the reviewer's commitment to improving the manuscript by clarifying and improving the accuracy of the results. We accept the reviewer's suggestions and make necessary modifications.

Modification 2.0
The respective modifications for each comment are indicated below.

Comment 2.1

This is regarding my original comment 2.10 which discusses the number of particles used in the simulation. It is not clear to me that going from N_p=1000 to N_p=1200 constitutes a small change in the force profile, or that N_p=1200 "matches well" with N_p=1000. In fact, with N_p=1000, it seems that force decreases during the holding time in the indentation experiment, while with N_p=1200 and N_p=10000, the force is almost constant during the holding time (even though the value of constant force between N_p=1200 and N_p=10000 is quite different during the holding time).

*Without a full analysis of how various N_p would generate different values for the other parameters after the data is recalibrated, and an analysis of the range of the other parameter values that would result form changing N_p, you should only claim that N_p must be set from the beginning and that changing N_p requires recalibration. You should *not* attempt to say anything quantitative like: "for small variations in the number of particles, we could still continue with the same parameter values, but for a drastic change...", as your analysis is not able to fully show this.*

Response 2.1
We have rephrased to avoid quantitative claims. Indeed, if a different number of particles, N_p, is used, then a renewed phase separation and recalibration are necessary. We have now reworded this sentence and the text in the Supplementary Information (SI) to clearly indicate a qualitative rather than a quantitative comparison. The modifications in SI are highlighted in yellow.

Modification 2.1

(Results; indentation calibration section, paragraph discussing Np)
Old text:
"Thus, BIOPOINT accurately reproduces experimental force-time curves, identifies dominant mechanical parameters, and optimizes mechanical parameters to match experimental results, ensuring a biophysically accurate, experimentally validated framework. One should carefully choose the resolution of BIOPOINT, and thus the number of particles in a cell, Np. If Np is varied, we might require sample preparation, nuclear phase separation and recalibration for a quantitative comparison with experiments. However, a small variation in Np doesn't alter the indentation force-time curve significantly (SI, Fig. S7)."

New text:
"Thus, BIOPOINT reproduces the experimental force–time curve for the SKOV3 indentation dataset used for calibration, identifies dominant mechanical parameters, and yields an optimized parameter set for this fixed resolution (Np = 1000). Importantly, the resolution of BIOPOINT—and thus the number of particles in a cell, Np — must be fixed prior to calibration. If Np is changed, particle size changes, and the nuclear phase separation should be redone. This requires re-equilibration and re-calibration before any quantitative comparison with experiments. We include Fig. S7 as a qualitative illustration that changing Np can affect the indentation response."

We have removed the following sentence from SI:

"~~Thus, for small variations in the number of particles, we could still continue with the same parameter values, but for a drastic change, a recalibration is advisable to quantitatively capture nonlinear deformation.~~"

Additional changes in SI,

Old text:
"We found that the elasticity is well reproduced for both Np =1200 and 10000, with a good agreement in the peak of the force-displacement curve at the end of the loading period of indentation. However, the force relaxation in the hold period for Np = 1200 matches well with Np = 1000, but the same is not true for Np = 10000."

New text:
"We found that the elasticity is qualitatively similar for both Np =1200 and 10000, with similar peaks of the force-displacement curve at the end of the loading period of indentation. The force relaxation in the hold period varies more dramatically for Np = 1200 and more so for Np = 10000."

Old text:
"We recommend that the particle size should be chosen carefully before using our model, depending on the length scale that one wishes to probe in their applications."

New text:
"We recommend that the particle number, and hence size, should be chosen carefully before using our model, depending on the length scale that one wishes to probe in their applications. If the particle number is changed, it is necessary to redo the phase separation, and recalibrate."

Comment 2.2

This is regarding my original comment 2.12 in which I asked about the the cell types you used to fit the numerical SEM experiments. You clarified in your response that the calibration was done only on the indentation experiments with SKOV3 and that the inferred parameters from the indentation experiments were then used for the spreading and migration simulations. You also mentioned that the later comparisons were with other cell types even though the model was calibrated with SKOV3.

*This needs to be made clear in the sections "Validation of BIOPOINT via cell spreading on ECM patterns" and "Validation of BIOPOINT in nuldear Deformation during constrained migration", and not just in the "Discussion" section. As the manuscript currently stands, the Results sections "Validation of BIOPOINT..." are hard to follow simply because you do not state clearly that you perform simulations using parameters calibrated on SKOV3 cell-indentation experiments only. You need to mention this, and you need to mention very explicitly that you now compare these new simulations (spreading, migration) to experiments in *other* cell types (EC and hMSC), so that the experimental data and the simulations are not expected to match, as the calibration was done with SKOV3. The data and simulations are only expected to have some qualitative correspondence. This sets up the correct expectation for the reader.*

*Moreover, in these sections, you should get rid of language like "successfully recapitulated", "matched experiments", "matched experimental results", and "consistent with experimental observations"; as the cell types are different, you cannot "recapitulate" or "match" anything, and this language is confusing. You should mention only qualitative agreement, things trending in the same direction, etc, as quantitative agreement is *not* expected. You should also mention the cell types EC and hMSC in the *text* of "Validation of BIOPOINT via cell spreading" and not just in the figure legends. If possible, you can incorporate your discussion of how parameters can differ between different cell types and how much difference you think exist between SKOV3 (which you calibrated on) versus EC and hMSC (which you compare to the other simulations).*

Response 2.2
We revised Results and Discussion to (i) explicitly state calibration is performed on SKOV3 indentation, (ii) clarify that comparisons to EC/hMSC and MDA-MB-231 datasets are qualitative benchmarks (no refitting), and (iii) remove overclaims and soften language ("matched", "validated", "in good agreement") where it implied cell-type–agnostic quantitative agreement. Salient changes are highlighted.

Modification 2.2

- **(Abstract; sentence starting "To ensure biological relevance…")**
  Old text:
  "To ensure biological relevance, we calibrate BIOPOINT against single-cell indentation experiments. We then validate the framework across three independent scenarios: (i) single-cell indentation with force–time responses matching atomic force microscopy data; (ii) cell spreading on ECM micropatterns, where nuclear deformation follows ECM geometry; and (iii) confined migration, where BIOPOINT reproduces nuclear shape dynamics as cells traverse constrictions."
  New text:
  "To ensure biological relevance, we calibrate BIOPOINT against single-cell indentation experiments (SKOV3). We then apply this calibrated parameter set, without additional refitting, to two independent scenarios: (i) cell (EC and hMSC) spreading on ECM micropatterns, capturing qualitative coupling between cell and nuclear shape; and (ii) confined migration (MDA-MB-231) through rigid constrictions, qualitatively reproducing the characteristic sequence of nuclear elongation and partial recovery."

- **(Results; Old subheading):**
  "Validation of BIOPOINT via Cell Spreading on ECM Patterns"
  New subheading:
  "Qualitative comparison: Cell Spreading on ECM Patterns"

- **(Results; Old subheading):**
  "Validation of BIOPOINT in Nuclear Deformation During Constrained Migration"
  New subheading:
  "Qualitative comparison: Nuclear Deformation During Constrained Migration"

- **(Results; cell spreading section opening paragraph)**
  Old text:
  "Having calibrated BIOPOINT's parameters, we next validated its ability to predict cell and nuclear shape evolution during ECM-guided spreading. … First, we want to assess whether BIOPOINT reproduces experimentally observed cell shape evolution on different ECM geometries…"
  New text:
  "Having calibrated BIOPOINT's mechanical parameters on SKOV3 indentation data (Fig. 2), we next assessed the model's predictions for cell and nuclear shape evolution during ECM-guided spreading using the same parameter set without additional refitting. … First, we assess whether BIOPOINT captures the qualitative ordering of cell shape evolution on different ECM geometries. Second, we quantify nuclear shape changes and compare qualitative NSI–CSI trends to published micropattern datasets (EC and hMSC; Fig. 3G), noting these are different cell types than the calibration dataset. Third, we isolate key parameters governing nuclear deformation."

- **(Results; spreading-results paragraph beginning "BIOPOINT simulations successfully…")**
  Old text:
  "BIOPOINT simulations successfully recapitulated cell spreading behaviors across ECM geometries… These trends align with experimental findings… The overall NSI trend versus CSI (Fig. 3G) matched experiments…"
  New text:
  "Using the SKOV3-calibrated parameter set (Fig. 2) without refitting, BIOPOINT reproduced the qualitative ordering of cell spreading across ECM geometries. Cells remained most circular on circular patterns (CSI near 1; Fig. 3E) and became progressively more elongated on square/triangular and then rectangular patterns (lowest CSI; Fig. 3E), consistent with published micropattern experiments on EC and hMSC cells [29,30]. Across these geometries, BIOPOINT predicted limited nuclear shape changes on isotropic patterns (circle, square, triangle; Fig. 3F) and modest nuclear elongation on elongated rectangles (decreased NSI; Fig. 3F). When compared to published EC and hMSC datasets (Fig. 3G), the model captures the qualitative coupling between cell shape (CSI) and nuclear shape (NSI) but underestimates the most extreme nuclear elongations reported for those cell types."
- **(Results; migration-results paragraph starting "Using the nuclear shape index…")**
  Old text:
  "… nuclear deformation matched experimental results (Fig. 5B). … consistent with experimental observations …"
  New text:
  "Using the nuclear shape index (NSI), we tracked nuclear shape over time and observed a deformation sequence that is qualitatively comparable to the published constricted-migration dataset of Keys *et al.* [34] (MDA-MB-231; Fig. 5B): the nucleus is rounded before entry (t_1), elongates during passage (t_2), and partially recovers after exit (t_3) (Fig. 5C–D; SV5). These simulations use the SKOV3-calibrated mechanical parameters without refitting; we therefore interpret this comparison as a qualitative benchmark rather than a cell-type-specific quantitative fit. BIOPOINT's particle-level representation enables spatial stress analysis

across the nucleus (Fig. 5E–F), illustrating how increasing nuclear stiffness increases nuclear stresses during transit. These results are consistent with experimental evidence that nuclear mechanics can influence migration efficiency in constrained environments [34].”

- **(Discussion; first paragraph, first sentence)**
  Old text:
  “Our simulations demonstrate that … align with experimental observations [28–32].”
  New text:
  “Our simulations reproduce the calibrated indentation force–time response and capture qualitative trends in cell spreading and confined migration observed experimentally [31–35].”
- **(Discussion; paragraph starting “BIOPOINT’s validation across multiple experimental conditions…”)**
  Old text:
  “BIOPOINT’s validation across multiple experimental conditions supports… We calibrated parameters on a particular cell type and cross-validated it across different cell types…”
  New text:
  “BIOPOINT is calibrated on a single cell-type dataset (SKOV3 indentation) and then benchmarked qualitatively against published datasets from other cell types where matched calibration datasets were not available. The qualitative agreements (e.g., Fig. 3G, Fig. 5B–D) suggest that some emergent trends are robust, but cell-type–specific quantitative predictions will require calibration and validation on matched datasets, since mechanical properties vary across cell types and conditions [44–46].”

Comment 2.3

You have not specified how u_0(1-2) and u_0(1-1) in Eq. (4) generalize from Eq. (3d), for example, to get u_0(1-2), do you need to replace 2R_cell with R1+R2?

Response 2.3
We clarified how Eq. (3d) generalizes to interactions between different particle types by defining the equilibrium spacing as the sum of the particles' radii (R_1 + R_2). We also corrected the description around Eq. (4) to consistently identify which particle types correspond to cytoplasm–cytoplasm and cytoplasm–nucleus interactions.

Modification 2.3
(Results; paragraph immediately following Eq. (4))
Old text:
"In Eq. 4, $u_0(1-2)$ and $u_0(1-1)$, respectively, represent potential well depth for the cytoplasm-ECM and cytoplasm-cytoplasm particle interactions, 1 and 2 representing particle type, as denoted by the index *j* in Eq. (1)."
New text:
"In Eq. 4, $u_0(1,2)$ and $u_0(1,1)$, represent the potential well depths for the cytoplasm–nucleus (types 1,2) and cytoplasm–cytoplasm (types 1,1) interactions, respectively. Eq. (3d) generalizes to interactions between particle types p and q as $u_{0(p,q)} = \kappa_{(p,q)} \frac{d^2_{eq(p,q)}}{(8\rho^2)}$, where $d_{eq(p,q)}$ is set by the equilibrium spacing of the two particle radii (e.g., $d_{eq(p,q)} = R_p + R_q$). In BIOPOINT, cytoplasmic and nuclear particles are equal-sized, so $d_{eq(1,2)} = d_{eq(1,1)}$, and we set $u_0(1,2) / u_0(1,1) = \kappa_{(1,2)} / \kappa_{(1,1)} = k_{12}$"

Comment 2.4

The colors of the graphs in Fig. 2D, 5F are hard to tell apart. It's hard to see the difference between blue and green.

Response 2.4
We have changed the colors of the graphs and also used different line styles in Fig. 2D and 5F to make them clearer. See the Modification section below.

Modification 2.4
We have modified the colors and line styles of the plot for better visibility.
Old Fig. 2D:

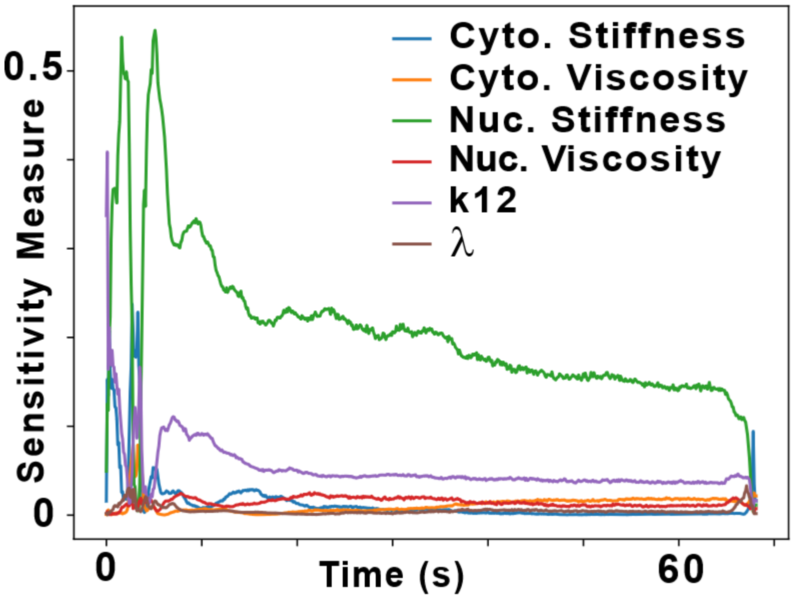

New Fig. 2D:

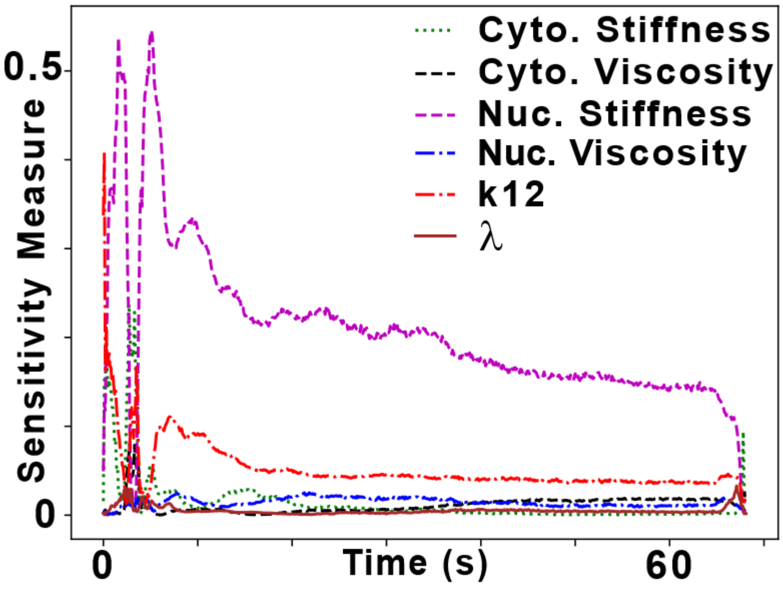

Old Fig. 5F:

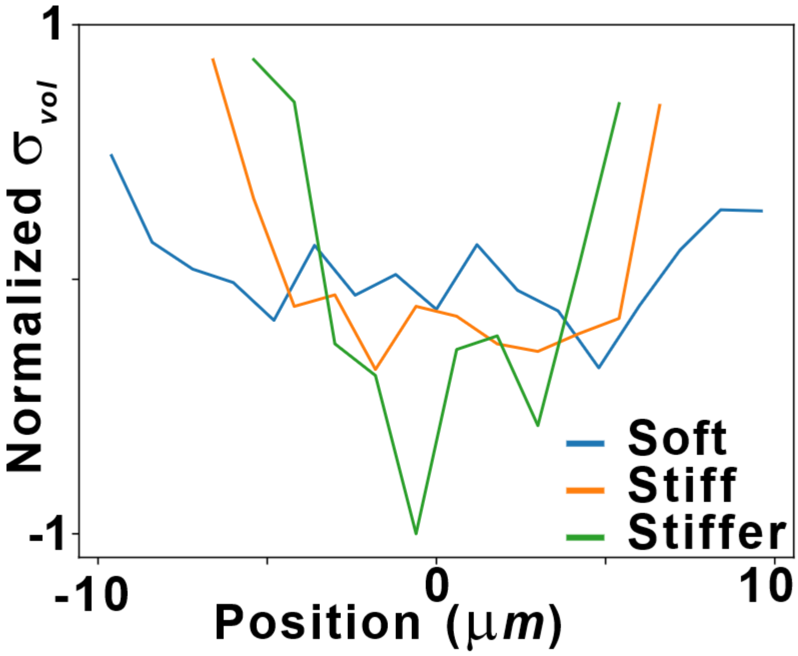

New Fig. 5F:

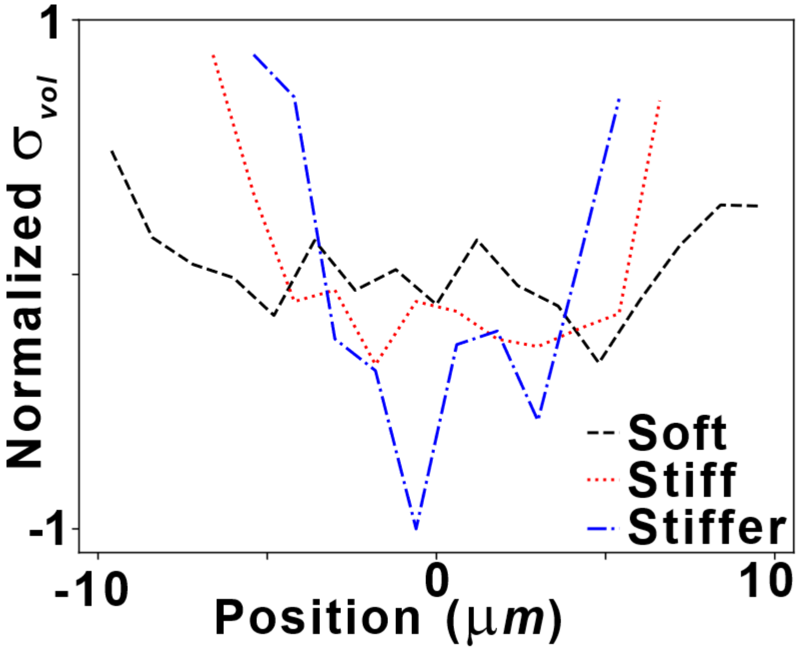

Comment 2.5

Fig 4C does not need the filling in the graph.

Response 2.5
We have removed the filling of the graph in Fig. 4C.

Modification 2.5

Old Fig. 4C:

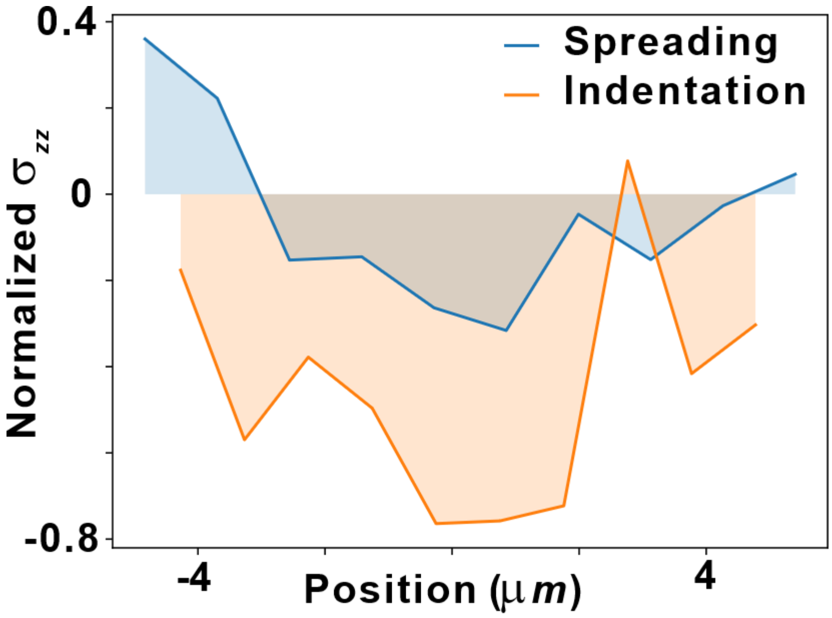

New Fig. 4C:

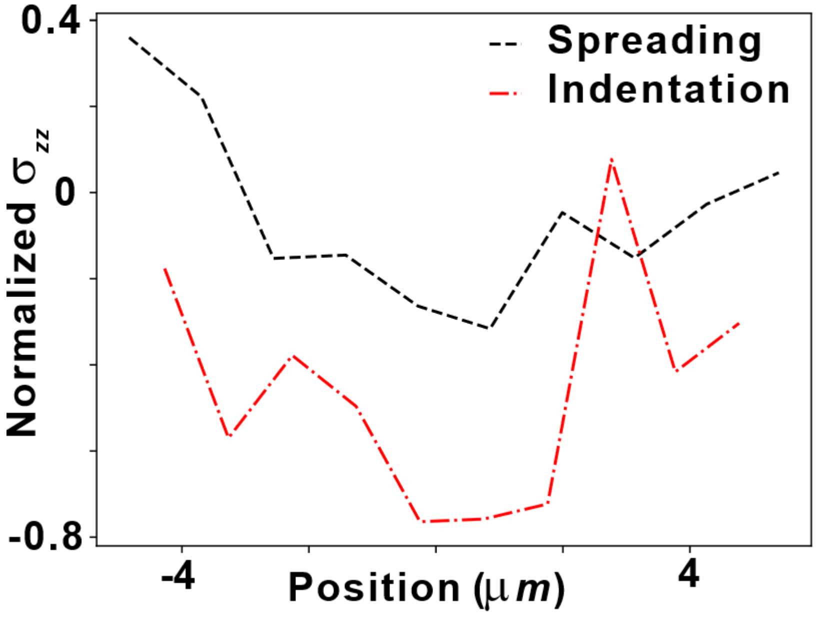

Supplement: Attachment — Submitted filename: BIOPOINT_R2_Response to reviewers.pdf [file pcbi.1014113.s009.pdf]
